# The tem­per­ature-dependent conformational ensemble of SARS-CoV-2 main protease (M^pro^)

**DOI:** 10.1107/S2052252522007497

**Published:** 2022-08-17

**Authors:** Ali Ebrahim, Blake T. Riley, Desigan Kumaran, Babak Andi, Martin R. Fuchs, Sean McSweeney, Daniel A. Keedy

**Affiliations:** a Diamond Light Source, Harwell Science and Innovation Campus, Didcot, OX11 0DE, England, United Kingdom; b Structural Biology Initiative, CUNY Advanced Science Research Center, New York, NY 10031, USA; c Biology Department, Brookhaven National Laboratory, Upton, NY 11973, USA; d Center for BioMolecular Structure, NSLS-II, Brookhaven National Laboratory, Upton, NY 11973, USA; e National Virtual Biotechnology Laboratory (NVBL), US Department of Energy, Washington, DC, USA; f Department of Chemistry and Biochemistry, City College of New York, New York, NY 10031, USA; g PhD Programs in Biochemistry, Biology, and Chemistry, The Graduate Center–City University of New York, New York, NY 10016, USA; University of Auckland, New Zealand

**Keywords:** protein structure, protein flexibility, X-ray crystallography, allostery, SARS-CoV-2 main protease, temperature-dependent, COVID-19

## Abstract

The COVID-19 pandemic, instigated by the SARS-CoV-2 coronavirus, continues to plague the globe. The SARS-CoV-2 main protease, or M^pro^, is a promising target for the development of novel antiviral therapeutics. Previous X-ray crystal structures of M^pro^ were obtained at cryogenic tem­per­ature or room tem­per­ature only. Here we report a series of high-resolution crystal structures of unliganded M^pro^ across multiple tem­per­atures from cryogenic to physiological, and another at high humidity. We inter­rogate these data sets with parsimonious multiconformer models, multi-copy ensemble models, and isomorphous difference density maps. Our analysis reveals a perturbation-dependent conformational landscape for M^pro^, including a mobile zinc ion inter­leaved between the catalytic dyad, mercurial conformational heterogeneity at various sites including a key substrate-binding loop, and a far-reaching intra­molecular network bridging the active site and dimer inter­face. Our results may inspire new strategies for antiviral drug development to aid preparation for future coronavirus pandemics.

## Abstract 2

X-ray crystallography at variable tem­per­ature for SARS-CoV-2 M^pro^ reveals a com­plex conformational landscape, including a mobile metal at the catalytic dyad, mercurial conformational heterogeneity at various sites, and an intra­molecular network bridging the active site and dimer inter­face.

## 1. Introduction

COVID-19 is a global pandemic disease caused by severe acute respiratory syndrome coronavirus 2. SARS-CoV-2 is a highly infectious airborne respiratory virus which has caused over 580 million infections and over 6.4 million deaths worldwide as of August 2022. Over the past two years, several approaches to prevent and treat COVID-19 have been successfully developed, including new vaccines, monoclonal antibody treatments (Baum *et al.*, 2020 ▸), and repurposed existing therapeutics (Beigel *et al.*, 2020 ▸; Boras *et al.*, 2021 ▸). More recently, small-mol­ecule antiviral drugs have fortunately been approved for clinical use to combat COVID-19. Notwithstanding these successes, additional chemical tools to modulate the function of coronavirus proteins would aid in the preparation for future coronavirus pandemics.

A promising target for potential new antiviral drugs against SARS-CoV-2 is a chymotrypsin-like protease known by several names: non-structural protein 5, nsp5, 3C-like pro­tease, 3CL^pro^, main protease, or M^pro^. M^pro^ is part of a polyprotein encoded by the viral RNA genome. After being excized from the polyprotein by its own proteolytic activity, M^pro^ cleaves at no fewer than 11 sites in the polyprotein to generate individual functional proteins (V’kovski *et al.*, 2021 ▸) that help the virus replicate. Due to its importance to the SARS-CoV-2 life cycle, M^pro^ has been identified as a key target for COVID-19 drug design.

**Figure 1:**
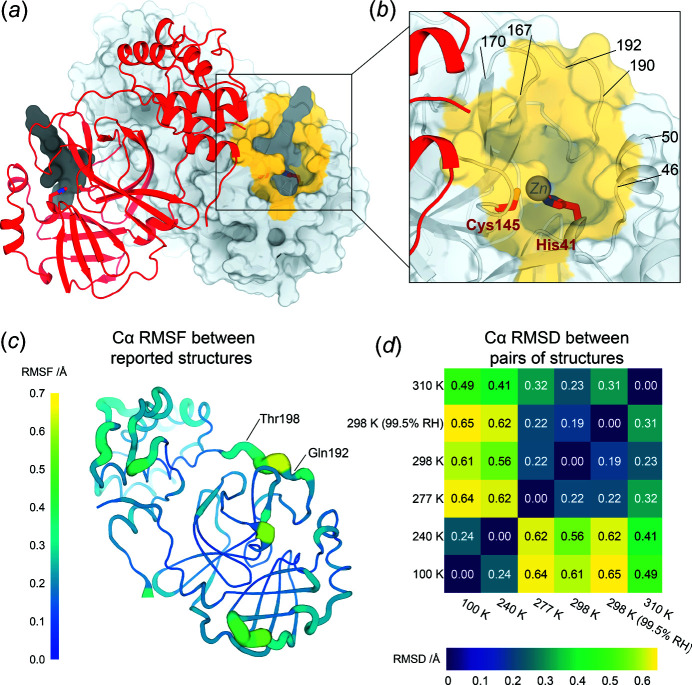
The overall structure of SARS-CoV-2 main protease at multiple tem­per­atures. (*a*) A new X-ray crystal structure of apo M^pro^ at physiological tem­per­ature (310 K) (red). The biological dimer involving the other monomer (light-gray surface) is constituted *via* crystal symmetry. The com­petitive inhibitor N3 from a previous structure (PDB entry 6lu7) (semitransparent, dark-gray surface) is shown in both protomers for context. (*b*) Close-up view of the M^pro^ active-site region, including the catalytic dyad of Cys145 and His41 (red sticks), and highlighting residues that form the substrate-binding pocket (yellow surface). (*c*) Cartoon putty representation of conformational variability between new M^pro^ structures described in this work: 100, 240, 277, 298, 298 (99.5% RH), and 310 K. Thickness and color indicate r.m.s. fluctuations (RMSFs) of the Cα-atom positions, from low (thin, dark blue) to high (thick, yellow). The largest differences between the backbones of these structures occur between residues 192–198. Same view as part (*a*). See also Fig. S3. (*d*) Heatmap of pairwise Cα-atom r.m.s. deviation (RMSD) between the final refined structures, revealing the tem­per­ature-dependent clustering (top right *versus* bottom left). See also Fig. S2.

Drug design efforts focused on M^pro^ have been aided by insights from structural biology. The first SARS-CoV-2 M^pro^ crystal structures were released in the Protein Data Bank (PDB; Berman *et al.*, 2000 ▸) early in the pandemic, within the first week of February 2020 ▸ (Jin *et al.*, 2020 ▸). These structures revealed that, like SARS-CoV M^pro^ before it, SARS-CoV-2 M^pro^ is com­posed of two β-barrel domains known as domain I and domain II, and an α-helical bundle known as domain III [Fig. 1 ▸(*a*)]. The active-site cavity is located on the surface, with the His41-Cys145 catalytic dyad positioned between domain I and domain II. Domain III is involved in regulating dimerization (Zhang *et al.*, 2020 ▸), which is critical for coronavirus M^pro^ catalytic activity (Fan *et al.*, 2004 ▸; Goyal & Goyal, 2020 ▸). Since the initial structures of SARS-CoV-2 M^pro^, X-ray crystallography has been used to identify promising ligand-binding sites and alternate structural states of the protein, resulting in a total of over 310 available structures. These efforts included cocrystallography with an eye toward drug repurposing (Vuong *et al.*, 2020 ▸; Günther *et al.*, 2021 ▸), as well as crystallographic screens of noncovalent and covalent small-mol­ecule fragments to establish new toeholds for *ab initio* drug design (Douangamath *et al.*, 2020 ▸), which were then leveraged *via* a crowdsourced process to design novel inhibitors (Chodera *et al.*, 2020 ▸).

As with much modern protein crystallography, the above experiments were performed at cryogenic tem­per­atures, which can bias protein conformational ensembles (Fraser *et al.*, 2011 ▸; Keedy *et al.*, 2014 ▸). To bypass this limitation, a room-tem­per­ature crystal structure of unliganded M^pro^ was reported (Kneller, Phillips, O’Neill, Jedrzejczak *et al.*, 2020 ▸) (PDB entry 6wqf), although its resolution was only moderate (2.3 Å) and the conclusions of the article about the effects of cryogenic *versus* room tem­per­ature were later questioned (Jaskolski *et al.* 2021 ▸). Subsequent work built on this foundation of room-tem­per­ature crystallography to dissect M^pro^ function (Kneller, Phillips, Kovalevsky *et al.*, 2020 ▸; Kneller, Phillips, O’Neill, Tan *et al.*, 2020 ▸). However, no studies to date have reported crystal structures of M^pro^ across a wide range of tem­per­atures. Previously, such a multitem­per­ature crystallography strategy was instrumental for revealing novel aspects of correlated active-site conformational heterogeneity in a dynamic proline isomerase (Keedy *et al.*, 2015 ▸) and of long-range allosteric signaling in a therapeutic target tyrosine phosphatase (Keedy *et al.*, 2018 ▸).

Here we report high-resolution crystal structures of SARS-CoV-2 M^pro^ at five tem­per­atures: 100 (cryogenic), 240 (above the so-called glass transition or dynamical transition; Keedy *et al.*, 2015 ▸), 277 (‘room tem­per­ature’ in many crystallography studies), 298 (ambient), and 310 K (physiological). We also report a structure at ambient tem­per­ature but high relative humidity (99.5% RH) to gauge the relative effects of tem­per­ature *versus* humidity on M^pro^. To our knowledge, this study represents the first experimentally based structural analysis for any SARS-CoV-2 protein at variable tem­per­ature and/or humidity. We used careful data collection with a helical strategy to minimize radiation damage, thereby isolating the effects of tem­per­ature and humidity on M^pro^. For all data sets we have constructed parsimonious multiconformer models, as well as multi-copy crystallographic ensemble models, which provide com­plementary insights into protein structural flexibility as a function of tem­per­ature and humidity. Together, our data reveal a network of subtle but provocative tem­per­ature-dependent conformational heterogeneity spanning several functionally relevant sites throughout M^pro^, which may help motivate an allosteric strategy for antiviral drug design to aid the preparation for future coronavirus pandemics.

## 2. Results

### 2.1. Multitem­per­ature crystallographic data collection and modeling

Data were obtained from single M^pro^ crystals using helical data collection, to maximize diffraction intensity while minimizing radiation damage (Fig. S1 in the supporting information). To probe the conformational landscape of M^pro^, we obtained high-resolution structures at five different tem­per­atures: 100, 240, 277, 298 (ambient; see *Methods* section), and 310 K. Our data sets thus span a broad tem­per­ature range: cryogenic, just above the glass transition or dynamic transition (Keedy *et al.*, 2015 ▸), the range often noted as room tem­per­ature (roughly 293–300 K), and approximately physiological tem­per­ature. We also collected another 298 K data set with high relative humidity (99.5% RH).

For all but the 277 K data set (2.19 Å), the resolution was 2 Å or better [based on an outer shell CC_1/2_ cut-off of >∼0.3 (Karplus & Diederichs, 2012 ▸); see *Methods* section and Table 1 ▸]. The highest resolution was for the 240 K data set (1.53 Å). Even at the higher tem­per­atures, we saw little to no evidence of radiation damage (Fig. S1). After data reduction, we created a multiconformer model for each tem­per­ature, which includes a single con­former for most portions of the structure, but alternate con­formations where appropriate (Riley *et al.*, 2021 ▸). See *Methods* section for more details on data collection and modeling, and Table 1 ▸ for the overall diffraction data and refinement statistics.

### 2.2. Overall structure as a function of tem­per­ature

The global structure of M^pro^ in our crystals remains similar across the tem­per­atures [Figs. 1 ▸(*d*) and S2], as expected. Indeed, the maximum Cα r.m.s. deviation (RMSD) between any pair of structures in the ambient-humidity multitem­per­ature series is only 0.64 Å, and the maximum all-atom RMSD is only 0.95 Å. However, there is a clear clustering between lower-tem­per­ature (100 and 240 K) and higher-tem­per­ature (277, 298, and 310 K) structures, based on either Cα RMSD [Fig. 1 ▸(*d*)] or all-atom RMSD (Fig. S2). These observations indicate that aspects of the M^pro^ conformational landscape change in response to tem­per­ature.

Humidity also appears to have some effect on M^pro^ structure, as shown by the fact that the overall largest pairwise Cα RMSD [0.65 Å, Fig. 1 ▸(*d*)] and all-atom RMSD (1.02 Å, Fig. S2) involve the 298 K high-humidity (99.5% RH) structure. However, the corresponding RMSD values for the 298 K ambient-humidity (36.7% RH) structure are only slightly smaller (<0.1 Å difference). These RMSD differences between high *versus* low humidity are minor com­pared to the differences between the high- *versus* low-tem­per­ature clusters mentioned above. Thus, tem­per­ature affects the M^pro^ structure noticeably more than does humidity. This result contrasts with previous studies of lysozyme in which similar structural alterations of the protein were achieved by either small changes in humidity or large changes in tem­per­ature (Atakisi *et al.*, 2018 ▸); this discrepancy may result from different protein–solvent arrangements in the lysozyme *versus* M^pro^ crystal lattices.

### 2.3. Temperature dependence of local alternate conformations

To provide more detailed insights into the observed global tem­per­ature dependence, we sought to identify alternate conformations at the local scale that were stabilized or modulated by the tem­per­ature shifts in our experiments. We specifically focused our attention on areas of the protein that are of inter­est for drug design and/or biological function: the active site, nearby loops associated with substrate binding, and the dimer inter­face.

First, the M^pro^ active-site structure remains mostly consistent across our tem­per­ature series (Fig. S4). The catalytic amino acids are in very similar conformations across the tem­per­atures. Additionally, a key active-site water mol­ecule (known as H_2_O_cat_), which hydrogen bonds to His41 of the catalytic dyad and the side chains of His164 and Asp187 (both in the active site), remains in the same position across our structures (Fig. S4). It has been suggested (Kneller, Phillips, O’Neill, Jedrzejczak *et al.*, 2020 ▸) that this water may play the role of a third catalytic residue (in addition to the catalytic dyad of His41 and Cys145). As noted previously (Kneller, Phillips, O’Neill, Jedrzejczak *et al.*, 2020 ▸), H_2_O_cat_ is not modeled in some cryogenic structures – but it is modeled in 87% (272/311) of the publicly available structures of SARS-CoV-2 M^pro^ as of October 11, 2021 ▸ (the vast majority of which are cryogenic), and perhaps should have been modeled in others (Jaskolski *et al.*, 2021 ▸).

As with the active-site amino acids and H_2_O_cat_, a dimethyl sulfoxide (DMSO) mol­ecule from the crystallization solution is ordered nearby in each structure in the multitem­per­ature series (Fig. S4, left of each panel). Inter­estingly, however, this DMSO molecule is displaced by a water mol­ecule in the high-humidity data set (298 K, 99.5% RH) [Fig. S4(*f*)], suggesting that the solvation distribution of the M^pro^ active site is malleable. Similarly, another DMSO molecule in a distal region of the protein is ordered throughout the multitem­per­ature series, but two waters and a new side-chain rotamer for Arg298 displace it in the high-humidity data set.

In addition to these solvent mol­ecules, we observe an unanti­cipated feature in the active site in all of our data sets: an electron-density peak between the side chains of Cys145 and His41, which form the catalytic dyad (Figs. S4 and S5). We initially modeled a water mol­ecule at this position, as in two previous apo structures: 7k3t Version 1.0 (the highest-resolution M^pro^ structure available; apo state; Andi *et al.*, 2022 ▸) and 7jfq (‘de-oxidized C145’, no publication). However, the inter­atomic distances between a putative water oxygen and the nearest atoms in Cys145 and His41 are relatively short, leading to steric clashes (Fig. S6), so we explored other possible explanations for this peak. Zn^2+^ binds tightly to M^pro^ with 300 n*M* affinity (Panchariya *et al.* 2021 ▸), and when soaked into M^pro^ crystals in previous studies (PDB entry 7dk1 and 7b83) [Fig. S9(*b*)], it was well ordered at our site of inter­est amidst the catalytic dyad. By contrast, we did not intentionally include Zn^2+^ at any stage, and Zn^2+^ is absent from almost all structures of M^pro^ in the PDB aside from a select few structures for which it was intentionally included, arguing against its presence in our structures. Nonetheless, we considered the possibility that low levels of cellular Zn^2+^ serendipitously bound to our sample of M^pro^ during expression and purification, and remained present during crystallization and X-ray data collection.

**Figure 2:**
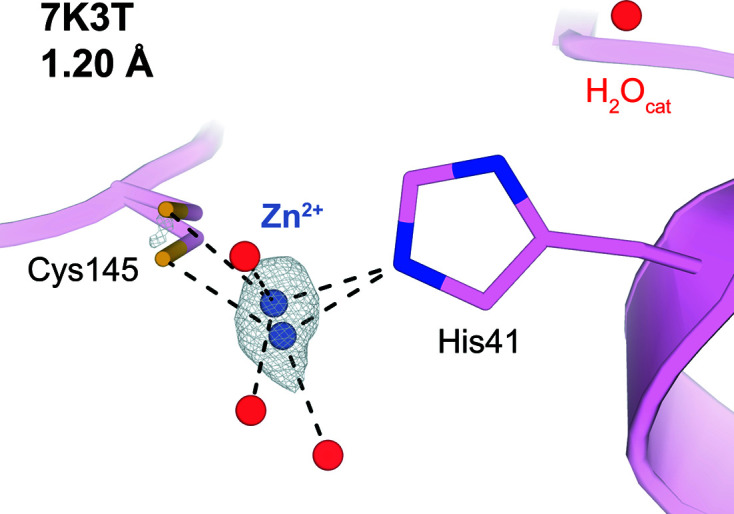
7k3t anomalous density map, contoured at 4σ. Anomalous density is only present in the asymmetric unit above 4 σ in the vicinity of the active site (as shown here). Both Zn^2+^ alternate conformations modeled in 7k3t display tetra­hedral coordination geometry, as shown with black dotted lines.

We therefore performed X-ray fluorescence (XRF) experiments on the original crystallization drops. The XRF results are consistent with the presence of Zn^2+^ (Fig. S7), but not other candidate metals, such as Ni^2+^. Although we did not observe significant anomalous density at this site, this may be due to the fact that our diffraction data had been collected at a wavelength that does not perfectly align with the Zn^2+^ anomalous edge (see *Methods* section). We performed XRF many months after initial diffraction data collection, at which time the crystals no longer diffracted, so we could not collect new anomalous diffraction data at the Zn^2+^ anomalous edge for the crystals constituting our multitem­per­ature series. Preparing a new batch of crystals to do so might have led to differences in the metal content, particularly given the absence of Zn^2+^ from the vast majority of structures of M^pro^. Instead, we re-examined the original diffraction data for the apo structure 7k3t, which – critically – were collected from the same batch of crystals as our multitem­per­ature data sets reported here, and are of significantly higher resolution (1.20 Å). We observe a strong anomalous peak at the position in question for 7k3t, despite also having used an off-edge wavelength for Zn^2+^ (Fig. 2 ▸). A new 7k3t Version 2.0 model is therefore deposited in the PDB with anomalous data included and Zn^2+^ instead of H_2_O modeled, and is described elsewhere (Andi *et al.*, 2022 ▸).

In response to these observations, we have modeled Zn^2+^ at partial occupancy (0.20–0.31) in each of our multitem­per­ature structures (Fig. S4). These refined crystallographic occupancies are similar and imply a small energy difference (Davis *et al.*, 2006 ▸) of <0.5 kT. The resulting structures have excellent Zn^2+^-binding geometry and the resulting maps are free of large difference peaks. Inter­estingly, in our tem­per­ature series, the position of Zn^2+^ varies across tem­per­atures by nearly 1 Å, which is in excess of the estimated coordinate error ranging from ±0.08 to ±0.32 Å for our structures (calculated using the Diffraction Precision Index online server; Kumar *et al.*, 2015 ▸), along an approximately linear swath (Fig. S8). This result is in line with the presence of alternate conformations displaced by over 0.7 Å along a similar vector for the Zn^2+^ in the higher-resolution 7k3t Version 2.0 [Fig. S9(*a*)], and also coincides with a swath of O atoms from a series of covalent ligands [Fig. S8(*c*)].

**Figure 3:**
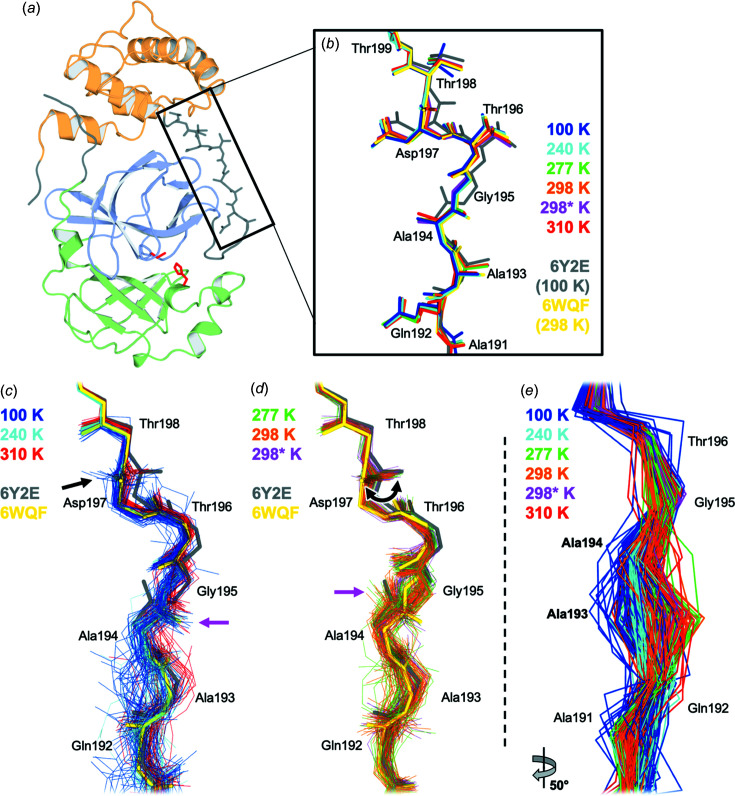
Complex tem­per­ature dependence of residues 192–198 in the P5 binding pocket. (*a*) M^pro^ monomer from a cryogenic structure, colored by domain: domain I, residues 8–101, pale green; domain II, residues 102–184, pale blue; domain III, residues 201–303, pale orange. Catalytic dyad residues Cys145 and His41 are shown as sticks (red). Terminal residues are shown in dark gray. P5 binding-pocket linker loop (residues 190–200) shown in dark gray and as sticks (black box). (*b*) Our new multitem­per­ature structures all have a single backbone conformation for this linker loop region. Regardless of tem­per­ature, they all match a similar backbone conformation to the room-tem­per­ature 6wqf model (yellow), and not the cryogenic 6y2e model (gray) (Kneller, Phillips, O’Neill, Jedrzejczak *et al.*, 2020 ▸) (298* K = 298 K, 99.5% relative humidity). (*c*)–(*e*) *Phenix* ensemble refinement models based on our multitem­per­ature data sets reveal a com­plex pattern of flexibility that was ‘hidden’ in part (*b*). (*c*) For some conditions (100 K, blue; 240 K, cyan; 310 K, red), the ensemble models generally match 6wqf, albeit with variability around the average conformation. For the Ala194–Gly195 peptide (pink arrow), these ensembles match 6wqf. For the Asp197–Thr198 peptide (black arrow), they match 6wqf. (*d*) For other conditions (277 K, green; 298 K, orange; 298* K, magenta), the ensemble models exhibit shifts away from 6wqf and toward 6y2e. For the Ala194–Gly195 peptide, these ensembles match 6y2e (pink arrow) instead of 6wqf. For the Asp197–Thr198 peptide, they adopt a swath of orientations (black curved arrow) bridging 6wqf and 6y2e. (*e*) A ∼50° counterclockwise-rotated view of all multitem­per­ature ensemble models, shown as Cα atoms only, illustrates the conformational clustering of the 100–240 K ensembles around residues 193–194 in the P5 binding pocket (bold text), while the 277–310 K ensembles occupy a broader swath of positions within this region.

Beyond the active site, we turned our attention to the nearby P5 binding pocket, specifically the loop com­posed of residues 192–198 (Fig. 3 ▸). Previously, the first report of a room-tem­per­ature structure of M^pro^, which was in the apo form (PDB entry 6wqf) (Kneller, Phillips, O’Neill, Jedrzejczak *et al.*, 2020 ▸), noted that this loop adopted a different conformation than in a prior 100 K apo structure (PDB entry 6y2e) (Zhang *et al.*, 2020 ▸), including rotated peptide orientations for Ala194–Gly195 and Asp197–Thr198. However, all of the structures in our multitem­per­ature series, including at lower tem­per­atures (100 and 240 K), have a single backbone con­form­ation in this region that matches that of 6wqf [Fig. 3 ▸(*b*)]. In addition, other apo cryogenic structures, including one at high (1.2 Å) resolution (PDB entry 7k3t), also match the 6wqf backbone conformation. All of these structures (6wqf, 6y2e, 7k3t, and our multitem­per­ature series) derive from the same crystal form (Table 1 ▸). Thus, it appears that the different loop conformation adopted in 6y2e is not driven by tem­per­ature (Kneller, Phillips, O’Neill, Jedrzejczak *et al.*, 2020 ▸), nor by ligand binding or crystal-lattice effects, but rather by some other aspect of the crystallization details or sample-handling conditions – including, perhaps, idiosyncratic effects of crystal cryocooling (Halle, 2004 ▸; Keedy *et al.*, 2014 ▸). Our conclusion here is also supported by a recent retrospective analysis of existing structures (Jaskolski *et al.*, 2021 ▸).

### 2.4. Crystallographic ensemble models reveal distinct backbone conformational heterogeneity

We next aimed to com­plement this analysis of our manually built multiconformer models with a more automated and explicitly unbiased approach to modeling flexibility that can handle larger-scale backbone flexibility such as loop motions. Therefore, we turned to *Phenix* ensemble refinement, which uses mol­ecular dynamics simulations with time-averaged restraints to crystallographic data (Burnley *et al.*, 2012 ▸). *Phenix* ensemble models have been used fruitfully for many applications (Woldeyes *et al.*, 2014 ▸), including exploring the effects of tem­per­ature on protein crystals (Keedy *et al.*, 2014 ▸), assessing the conformational plasticity of peptide–MHC inter­actions (Fodor *et al.*, 2018 ▸), and rational protein design (Broom *et al.*, 2020 ▸). After a scan of parameter space (see *Methods* section), we created one ensemble model per tem­per­ature, each of which contains 28 to 75 constituent models (Table 2 ▸). Compared to the multiconformer models, the ensemble models fit the experimental data equally well or better based on *R*_free_, albeit with slightly wider *R*_free_–*R*_work_ gaps (Table 2 ▸ *versus* Table 1 ▸).

Using these ensemble models, we re-examined the P5 binding pocket loop mentioned above. The 100, 240, and 310 K ensemble models are similar to the previous ‘room-tem­per­ature’ structure 6wqf, with mostly the same peptide orientation for Ala194–Gly195 and Asp197–Thr198 [Fig. 3 ▸(*c*)]. By contrast, the 277, 298, and 298 K (99.5% RH) ensemble models mostly match the flipped Ala194–Gly195 peptide orientation from the previous cryogenic structure 6y2e, and sample a swath of conformations for Asp197–Thr198 that span 6wqf and 6y2e [Fig. 3 ▸(*d*)]. This distinction between peptide conformations that match 6wqf *versus* 6y2e is not simply a by-product of resolution, as 298 K is higher resolution than 310 K. More broadly, our ensembles reveal that the backbone of residues 192–195 occupies a distinct clustered conformation at the lower tem­per­atures of 100–240 K, while sampling a com­prehensive swath of positions at the higher tem­per­atures of 277–310 K [Fig. 3 ▸(*e*)]. Taken together, these results suggest that this region of M^pro^ has a com­plex relationship between tem­per­ature and conformational heterogeneity.

**Figure 4:**
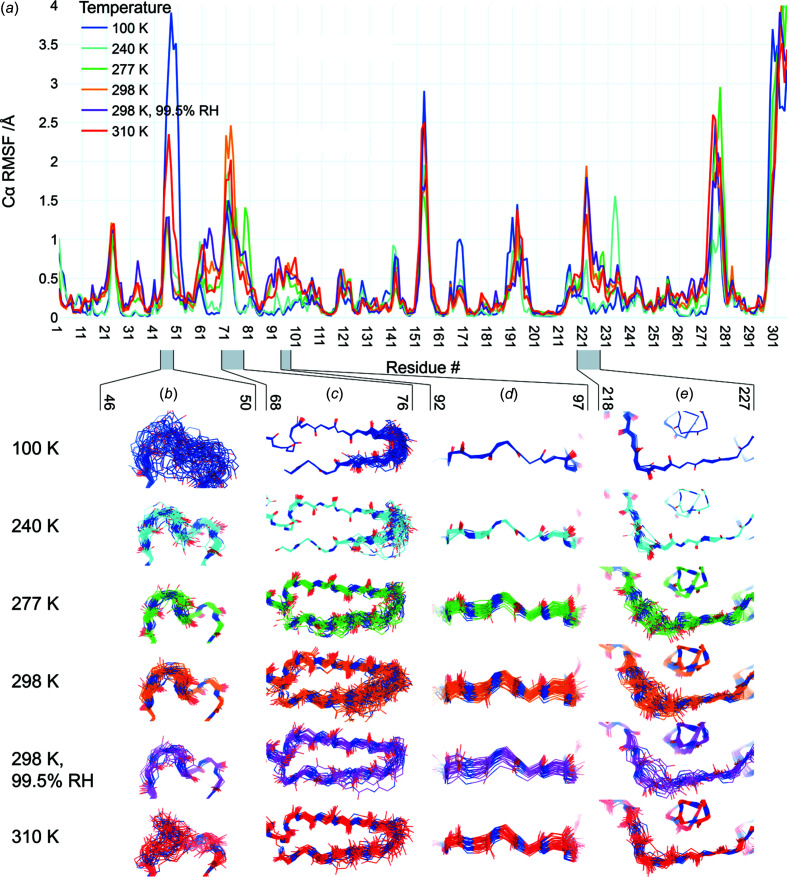
Backbone structural variability of ensemble models along the M^pro^ sequence as a function of tem­per­ature. (*a*) RMSF of the backbone Cα-atom positions is plotted *versus* residue number for each of the different structures in our multitem­per­ature series (colors in legend). RMSF spikes at the N-terminus, C-terminus, and β-turn 153–157 (in contact with the C-terminus) in the ensemble models are truncated in this plot, and should be inter­preted with caution. (*b*)–(*e*) Backbone structures from ensemble refinement are shown for regions coinciding with tem­per­ature-dependent RMSF peaks. The refined single structure is shown as a cartoon, while atoms in the backbone of ensemble models are shown as lines.

Beyond just the P5 loop, we also examined other regions with elevated and/or tem­per­ature-dependent ensemble Cα r.m.s. fluctuation (RMSF) [Fig. 4 ▸(*a*)] that were not previously noted as being tem­per­ature dependent. These regions segregate into different categories with distinct tem­per­ature depen­dence.

First, some regions display a generally positive correlation between backbone structural variability and diffraction ex­peri­ment tem­per­ature. For example, in residues 68–76 [Fig. 4 ▸(*c*)], conformational diversity is restricted to the β-hair­pin at 100 and 240 K, but appears to spread further down the β-strands at higher tem­per­atures. In another case, residues 92–97 [Fig. 4 ▸(*d*)] and 218–227 [Fig. 4 ▸(*e*)] are highly ordered at 100 and 240 K, but mobile at warmer tem­per­atures. Inter­estingly, although these two regions (92–97 and 218–227) are isolated from each other in the monomer and the biologically relevant dimer, together they form a contiguous patch with 68–76 in the crystal lattice. Finally, we observe one region with an atypical relation between backbone variability and tem­per­ature: the short 3_10_ helix at residues 46–51 [Fig. 4 ▸(*b*)]. This region abuts the P5 substrate-binding loop com­posed of residues 192–198 with its com­plex tem­per­ature dependence (Fig. 3 ▸); together these two regions form one side of the active-site pocket [Fig. 1 ▸(*b*)].

### 2.5. A network of coupled conformational heterogeneity bridges the active site, substrate pocket, interdomain inter­face, and dimer inter­face

**Figure 5:**
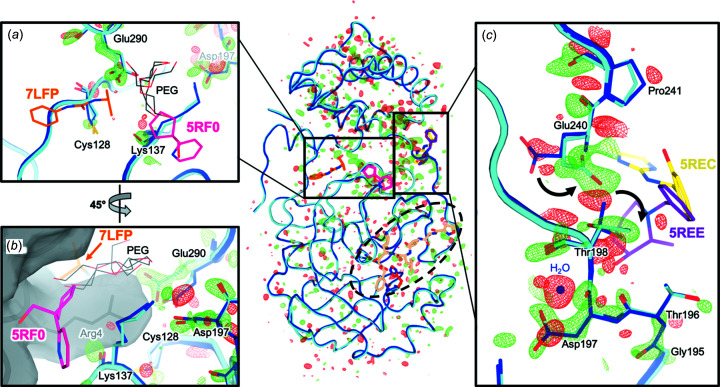
*F*_o_ − *F*_o_ difference maps reveal local conformational shifts connecting the active site, interdomain inter­face, and dimer inter­face. Center: overview of the isomorphous *F*_o_ − *F*_o_ difference electron-density map at ±3σ (green–red mesh) for the 240 K data set (cyan) minus the 100 K data set (dark blue) (see Fig. S10 for *F*_o_ − *F*_o_ maps for all tem­per­atures). Ligands from cocrystal structures are shown at the active site (dashed oval) (pale orange, 6lu7), interdomain inter­face (purple, 5ree; yellow, 5rec), and dimer inter­face (orange, 7lfp; pink, 5rf0). (*a*) Glu290 switches from one side-chain rotamer at 100 K to two alternate rotamers at 240 K. Glu290 is spatially adjacent to Cys128, which switches from two alternate rotamers at 100 and 240 K to a single rotamer at 277 K and above in our multiconformer models; the alternate rotamer occupancy is lower at 240 K, consistent with its positive *F*_o_ − *F*_o_ peak (see Fig. S12). These residues are near two ligands from separate crystallographic screens (7lfp and 5rf0), as well as many ordered PEG mol­ecules from the crystallization cocktails of various structures (7kvr, 7kvl, 7kfi, and 7lfe). (*b*) An ∼45°-rotated view relative to part (*a*) shows that these two ligands bind at the dimer inter­face of the biological monomer, constituted in the crystal from a symmetry-related protomer (gray surface). This inter­face also includes the Asp197 region (right). (*c*) Thr198 switches from two alternate side-chain rotamers at 100 K to a single rotamer at 240 K, while Glu240 – located across the interdomain inter­face – changes side-chain rotamer (curved arrows), with additional effects on the adjacent backbone of Pro241. In the other direction from Asp197 (down in this view), other residues in the P5 substrate-binding pocket loop (Fig. 3 ▸) undergo conformational adjustments en route to the active site. Meanwhile, an inter­acting water mol­ecule at 100 K (blue sphere) becomes displaced at 240 K, and is correspondingly absent in that model.

To com­plement the model-centric approaches above, we also looked for tem­per­ature-dependent conformational effects using an approach that is more directly data-driven: isomorphous *F*_o_ − *F*_o_ difference electron-density maps. We com­puted *F*_o_ − *F*_o_ difference maps for each tem­per­ature *versus* 100 K, and looked for patterns in terms of spatial colocalization of difference peaks. The global results confirm that the protein structure remains similar overall, with a smattering of difference peaks throughout the monomer asymmetric unit (Fig. S10). However, within those difference peaks lies a provocative stretch of difference features spanning the dimer inter­face, the inter­face between domain I and domain II of the monomer, and the edge of the P5 substrate-binding pocket (Fig. 5 ▸). These difference features may be somewhat resolution dependent, as they are least pronounced for 277 K (2.19 Å) and most pronounced for 240 K (1.53 Å), but their distribution across M^pro^ is qualitatively similar across tem­per­atures. These features are not an artifact of diffraction anisotropy (Fig. S11).

A closer examination of the models in the vicinity of these difference features reveals what appears to be a series of correlated conformational motions keyed to tem­per­ature change. For example, *F*_o_ − *F*_o_ density shows that Glu290 shifts from a single side-chain rotamer at 100 K to two alternate rotamers with partial occupancies at 240 K [Fig. 5 ▸(*a*)]; this second rotamer seen at 240 K then remains as a single full-occupancy conformation for all higher tem­per­atures. Spatially adjacent to Glu290, Cys128 gradually shifts from two alternate rotamers at 100 K toward a single rotamer at higher tem­per­atures; at the inter­mediate tem­per­ature of 240 K, the alternate rotamer still exists but has lower occupancy than at 100 K (Fig. S12), which is consistent with the presence of a positive 240–100 K *F*_o_ − *F*_o_ peak [Fig. 5 ▸(*a*)]. Both Glu290 and Cys128 inter­act with a symmetry-related Arg4 across the biological dimer inter­face [Fig. 5 ▸(*b*)]. Inter­estingly, two small-mol­ecule fragments from recent crystallographic screens (Douangamath *et al.*, 2020 ▸; Noske *et al.*, 2021 ▸) bind at this area of the dimer inter­face [Figs. 5 ▸(*a*) and 5(*b*)]. Moreover, ordered polyethyl­ene glycol (PEG) mol­ecules from several previous structures [PDB entries 7kvr, 7kvl, 7kfi, and 7lfe; Figs. 5 ▸(*a*) and 5(*b*)] illustrate the potential for future ligand design efforts to ‘grow’ from one of these initial fragment hits (5rf0) toward the mobile Glu290 and Cys128. This observation reinforces the idea that mol­ecules from crystallization solutions, such as glycols, can reveal useful features like cryptic binding pockets (Bansia *et al.*, 2021 ▸).

Glu290 is connected to another inter­esting residue, Asp197, *via* a hydrogen-bond network with only one inter­vening side chain (Arg131). Within this vicinity, an inter­acting water mol­ecule is liberated, and an adjacent residue, Thr198, shifts from two alternate side-chain rotamers to just one [Fig. 5 ▸(*c*)]. The Thr198 motion is linked to a conformational re-ordering for the nearby Glu240 side chain and Pro241 backbone, thus establishing a possible means for allosteric communication across the interdomain inter­face. In the opposite direction from Asp197, other adjacent residues experience changes in ordering per *F*_o_ − *F*_o_ peaks; these residues together form the 192–198 loop of the functionally important and mobile P5 pocket (Fig. 3 ▸) leading toward the active site.

Overall, these observations describe a series of conformational motions that bridge the dimer inter­face, interdomain inter­face, substrate-binding pocket, and active site (Fig. 5 ▸, center, boxes and oval). In this work, tem­per­ature is the per­tur­bation/effector – but our results raise the enticing pos­sibility that future small mol­ecules could be used to allo­sterically perturb this network, thereby modulating enzyme dimerization and/or catalysis.

## 3. Discussion

Our crystal structures of unliganded SARS-CoV-2 M^pro^ at variable tem­per­ature and humidity paint a picture of a com­plex protein conformational landscape. The structure of M^pro^ does not change linearly with tem­per­ature; rather, there is a global transition between roughly <240 and >277 K [Figs. 1 ▸(*d*) and S2]. This 240–277 K transition regime for M^pro^ does not coincide with the 180–220 K glass transition or dynamical transition threshold seen previously for other systems such as CypA (Keedy *et al.*, 2015 ▸), suggesting protein-to-protein variability. More locally in M^pro^, as tem­per­ature increases, different regions experience distinct types of changes to conformational heterogeneity (Fig. 4 ▸), in line with previous multitem­per­ature studies of other proteins (Keedy *et al.*, 2014 ▸). These effects are not limited to surface-exposed side chains as one might naïvely expect, but rather encom­pass motions of buried side chains (Fig. 5 ▸) and many backbone regions (Figs. 3 ▸ and 4 ▸). Our results here for M^pro^, as well as a large body of previous literature for other systems, refute the assertion that X-ray crystallography under ‘unusual experimental conditions’ like variable tem­per­ature is not useful for understanding proteins (Jaskolski *et al.*, 2021 ▸). By contrast, our work is in line with com­putational analyses of *B* factors suggesting that different alternate conformations for M^pro^ (and other systems) can be accessed by varying tem­per­atures and/or the crystal lattice (Pearce & Gros, 2021 ▸).

Our structures contain serendipitously bound low-occupancy Zn^2+^ at the site of chemistry, presumably from the bac­terial cells used for protein expression. It should be noted that the presence of Zn^2+^ in the active site of our structures may bias the tem­per­ature response and com­plicate efforts to exploit these structures for structure-based drug design. It is unclear why this Zn^2+^ is absent in most previous structures of M^pro^. Notably, recent structures of an acyl–enzyme inter­mediate structure (PDB entry 7khp) and a C145A mutant product-bound structure (PDB entry 7joy) of M^pro^ include a nearby water, ∼1.5 Å away but aligned with our approximately collinear multitem­per­ature Zn^2+^ swath, which the authors suggested may play a role as a de­acyl­ating nucleophile (Lee *et al.*, 2020 ▸). Questions about the energetic landscape of this catalytic dyad region and its relation to function could be explored in parallel with other experiments, such as variable pH to probe Cys145 oxidation and reactivity (Kneller, Phillips, O’Neill, Tan *et al.*, 2020 ▸), and neutron crystallography to reveal a zwitterionic state of the catalytic dyad (Kneller, Phillips, Weiss *et al.*, 2020 ▸), although questions remain about the inter­pretation of such data (Jaskolski *et al.*, 2021 ▸).

High relative humidity during data collection does not dramatically affect our structures [Figs. 1 ▸(*d*) and S2]. However, it does alter the solvation shell in the active site (Fig. S4, bottom left) and elsewhere. Displaceable waters could potentially be exploited to design high-affinity small-mol­ecule inhibitors, particularly when guided by water thermo­dynamics maps from simulations, as are available for M^pro^ and other SARS-CoV-2 proteins (Olson *et al.*, 2020 ▸). More broadly, this result hints at the utility of humidity as an experimental variable in crystallography (Kiefersauer *et al.*, 2000 ▸; Sanchez-Weatherby *et al.*, 2009 ▸) for exploring solvent slaving to solvent energetics in ligand binding (Darby *et al.*, 2019 ▸), protein dynamics (Lewandowski *et al.*, 2015 ▸), and other functionally relevant phenomena.

*Phenix* ensemble models (Burnley *et al.*, 2012 ▸) refined from our X-ray data sets helped us to illuminate tem­per­ature-dependent differences in conformational heterogeneity in certain areas of M^pro^ (Figs. 3 ▸ and 4 ▸) that were concealed by more traditional model types (Babcock *et al.*, 2018 ▸). Despite its utility in this and other work, there is significant potential for improvement of the ensemble refinement methodology through, for example, integration of more sophisticated mol­ecular mechanics force fields like Amber (Moriarty *et al.*, 2020 ▸) into the mol­ecular dynamics com­ponent (Burnley *et al.*, 2012 ▸) to improve ensemble model geometry, or more sophisticated treatments of translation–libration–screw (TLS) groups to isolate inter­esting local conformational heterogeneity (Ploscariu *et al.*, 2021 ▸). Although it was also beyond the scope of this study, ensemble models may reveal alternate conformational substates that are important for the catalytic cycle, which could be fruitfully targeted by small mol­ecules for antiviral drug design.

Finally, our results emphasize the allure of allosteric inhibition of M^pro^ as an alternative therapeutic strategy. Allosteric inhibitors hold the potential to target unutilized sites, though they can face mutational escape by the protein target (Lu *et al.*, 2020 ▸). Our structures illustrate apparently coupled conformational motions that bridge the active site, substrate-binding pocket, interdomain inter­face, and parts of the broad dimer inter­face (Figs. 4 ▸ and 5 ▸). This is particularly noteworthy since M^pro^ must dimerize to become an active enzyme (Fan *et al.*, 2004 ▸; Goyal & Goyal, 2020 ▸). Interdomain flexing has also been observed, even in crystals (Jaskolski *et al.*, 2021 ▸). The intra­molecular network we describe includes several sites that are distal from the active site, one of which is highlighted by Glu240 difference density [Fig. 5 ▸(*c*)] corresponding to a tem­per­ature-dependent rotamer flip, with this site having already been characterized as ligandable by recent crystallographic screens of pre-existing drug mol­ecules (Günther *et al.*, 2021 ▸) and small-mol­ecule fragments (Douangamath *et al.*, 2020 ▸) (Fig. 5 ▸). Some new M^pro^ ligands have been shown by mass spectrometry to disrupt the M^pro^ dimer and allosterically inhibit catalysis, albeit weakly thus far (El-Baba *et al.*, 2020 ▸), illustrating the potential of an allosteric strategy. As a com­plementary structure-based approach to current experiments on the dimeric crystal form of M^pro^, future experiments could exploit mutations of the dimer inter­face to stabilize an inactive monomer, thus capturing a new structural target for crystallographic and solution screening for allosteric inhibitors that block dimerization. Ultimately, the present study offers insights into fundamental aspects of protein structural biophysics, and may also help pave the way for new efforts toward allosteric modulation of M^pro^ as a strategy for coronavirus drug design.

## 4. Methods

### 4.1. Cloning, expression, and purification

Full details of the cloning, expression, and purification are reported elsewhere (Andi *et al.*, 2022 ▸). Briefly, the codon-optimized synthetic gene of full-length M^pro^ from SARS-CoV-2 was cloned into the pET29b vector. The cloned M^pro^ with C-terminal 6x histidine tag was expressed in *E. coli* using an auto-induction procedure (Studier, 2005 ▸). Cells were har­ves­ted, lysed using bacterial protein extraction agents (B-PER, ThermoFisher Scientific) in the presence of lysozyme, and purified with nickel-affinity chromatography followed by size-exclusion chromatography. The histidine tag was cleaved by human rhinovirus (HRV) 3C protease (AcroBIOSYSTEMS) and further purified by reverse nickel-affinity chromatography. The purified protein was then dialysed overnight at 4°C against 30 m*M* HEPES pH 7.4, 200 m*M* NaCl, 1 m*M* TCEP. Finally, the protein was concentrated to ∼7 mg ml^−1^ and either used for crystallization or stored at −80°C.

### 4.2. Crystallization

Plate-like crystals ranging from ∼100–400 µm along the longest axis (∼5–10 µm along the shortest axis) were grown *via* sitting-drop vapor diffusion. The crystals grew in ‘flower-like’ clusters (Fig. S13). After mixing a 1:1 ratio of ∼7 mg ml^−1^ M^pro^ with a solution of 22% PEG 4000, 100 m*M* HEPES pH 7.0, 3–5% DMSO and incubating at a tem­per­ature of ∼298 K, crystals were seen after 2–6 d.

### 4.3. Crystal harvesting and X-ray data collection

Individual crystals were harvested using 10 µm MicroMesh loops (MiTeGen). For cryogenic tem­per­ature, crystals were cryocooled by the traditional practice of plunging into liquid nitro­gen. For non-cryogenic tem­per­atures at ambient humid­ity, crystals were coated with Paratone-N oil, then mounted on the goniometer for data collection. Data sets were also collected for crystals coated with Paratone-N oil and additionally enclosed in MicroRT capillaries (MiTeGen), but no differences were observed relative to Paratone-N oil only. For high humidity, crystals were not coated with Paratone-N oil, but were enclosed in MicroRT capillaries for the short transit to the goniometer, then removed once a humid air flow was established on the goniometer; this ensured the crystal was always maintained at high humidity after leaving the crystallization drop. Each crystal was equilibrated on the goniometer for 10–20 min, which is more than sufficient to reach stable conditions.

X-ray diffraction data were collected at the National Synchrotron Light Source II (NSLS-II) beamline 17-ID-2 (FMX) (Schneider *et al.*, 2021 ▸) using an X-ray beam of energy 12.66 keV, corresponding to a wavelength of 0.9793 Å; a horizontal-bounce Si111 double-crystal monochromator; and an EIGER X 16M pixel array detector (Dectris). The temperature at the sample goniometer was controlled using a Cryostream 800 (Oxford Cryosystems). For the 298 K, 99.5% relative humidity (RH) data set, RH was controlled with an HC-LAB Humidity Controller (Arinax). Ambient tem­per­ature was measured to be ∼298 K and ambient humidity was measured to be 36.7%. A new crystal was used for each data set. Helical/vector data collection was used to traverse the length of each crystal, with a beam size of 10 × 10 µm. Using *RADDOSE-3D* (Bury *et al.*, 2018 ▸), we estimated the average diffraction-weighted dose (ADWD) for our data sets to be 242 kGy for 100 K, 532 kGy for 240 K, 397 kGy for 277 K, 137 kGy for 298 K, 182 kGy for 298 K (99.5% RH), and 176 kGy for 310 K. All of these ADWD values are at or below the estimated room-tem­per­ature limit of about 400 kGy (Fischer, 2021 ▸) for our higher tem­per­atures, although this limit is generally system dependent. The ADWD for 240 K is above the room-tem­per­ature limit, but such lower tem­per­atures have higher dose tolerance. Additionally, there was no evidence of global radiation damage from R_d_ plots (Fig. S1), and local/specific radiation damage did not appreciably accrue during the course of each single-crystal data collection, as indicated by 2*F*_o_ − *F*_c_ electron-density maps around carboxyl groups (not shown).

For independent verification of the presence of metals in the crystal samples, we collected X-ray fluorescence spectra and performed edge scans. For fluorescence spectra data collection, the radiation emitted and scattered from the crystal was detected with an energy-dispersive Si drift detector (Ketek VIAMP KC), in a configuration to collect radiation scattered or emitted in a horizontal direction perpendicular to the incoming X-ray beam. For energy edge scans, the incoming photon energy was scanned across the absorption edge of the scatterer under investigation while detecting the integrated intensity of photons in a region of inter­est around the expected associated X-ray fluorescence emission line.

### 4.4. X-ray data reduction and modeling

The data reduction pipeline *fast_dp* (Winter & McAuley, 2011 ▸) was initially used for bulk data reduction during the beamtime, with selected data reprocessed using the *xia2 DIALS* (Winter *et al.*, 2018 ▸) and *xia2 3dii* (*XDS* and *XSCALE*) pipelines (Kabsch, 2010 ▸), with *xia2 3dii* (*XDS* and *XSCALE*) also being used for the generation of R_d_ statistics (Diederichs, 2006 ▸) (Fig. S1). Resolution cut-offs were chosen based on CC_1/2_ being >∼0.3 in order to include data at lower signal levels that improve the model (Karplus & Diederichs, 2012 ▸), in combination with high overall and outer-shell com­pleteness (Afonine *et al.*, 2012 ▸; Arkhipova *et al.*, 2017 ▸). Mol­ecular replacement for each data set was performed *via Phaser-MR* from the *Phenix* software suite, using PDB entry 6yb7 as a search model. *Phenix AutoBuild* (Terwilliger *et al.*, 2008 ▸) was used for initial model building and refinement, with subsequent iterative refinements performed using *phenix.refine* (Afonine *et al.*, 2012 ▸) and *Coot* (Emsley & Cowtan, 2004 ▸). After a few initial rounds of refinements, H atoms were added using *phenix.ready_set* [*Reduce* (Word *et al.*, 1999 ▸) and *eLBOW* (Moriarty *et al.*, 2009 ▸)]. For refinement of each data set, X-ray/stereochemistry weight and X-ray/ADP weight were refined and optimized. Geometric and protein statistics of the final models were evaluated *via MolProbity* (Chen *et al.*, 2010 ▸; Williams *et al.*, 2018 ▸) and the JCSG–QC check server (https://smb.slac.stanford.edu/jcsg/QC/). Data collection and refinement sta­tistics are shown in Table 1 ▸.

Crystallographic ensemble models were generated using *phenix.ensemble_refinement* (Burnley *et al.*, 2012 ▸) in Version 1.18.2-3874 of *Phenix*. Alternate conformations were first removed from the multiconformer models, and H atoms were (re)added using *phenix.ready_set*. Next, a *phenix.ensemble_refinement* grid search was performed by repeating the simulation with four values of p_TLS_ (1.0, 0.9, 0.8, 0.6) and three values of wxray_coupled_tbath_offset (10, 5, 2.5), and using a random_seed value of 2679941. τ_x_ was set automatically accor­ding to the high-resolution limit of the data set. From this grid, we present the analysis of the set of ensemble models that has the lowest mean *R*_free_: p_TLS_ = 0.8 and wxray_coupled_tbath_offset = 10.0. Although we chose one ensemble model per data set, the trends we describe in this report were generally consistent across alternative ensemble models with different parameter choices. In line with this consistency, when com­paring the alternative ensemble models we considered, the *R*_free_ values for any particular crystal differed by at most 2.8% and *R*_work_ values by at most 1.6%. Refinement statistics are shown in Table 2 ▸.

For *F*_o_ − *F*_o_ isomorphous difference map analysis, the *phenix.fobs_minus_fobs_map* executable in the *Phenix* software suite was used. This program performs inter­nal scaling of the two data sets to each other. Each elevated tem­per­ature was com­pared to 100 K. The 100 K multiconformer model was used for phasing for each difference map. The effects of diffraction anisotropy were assessed using the *STARANISO* server (Tickle *et al.*, 2018 ▸) to perform an anisotropic cut-off of merged intensity data for all tem­per­atures. *STARANISO* data sets were com­pared to our multitem­per­ature series in *Coot* and using *phenix.fobs_minus_fobs* (Fig. S11) to survey whether diffraction anisotropy gives rise to variations in difference density. For solvent content analysis, *rwcontents* (Version 7.1.009) from the *CCP4* suite (Winn *et al.*, 2011 ▸) was used.

## 5. Accession numbers and data availability

Models and structure factors are available in the Protein Data Bank under the following PDB entry accession codes (see also Tables 1 ▸ and 2 ▸): 7mhf, 7mhg, 7mhh, 7mhi, 7mhj, and 7mhk for muticonformer models, and 7mhl, 7mhm, 7mhn, 7mho, 7mhp, and 7mhq for ensemble models. New versions (Version 2.0) of each of these structures were deposited after modeling Zn^2+^ instead of H_2_O in the active site.

Diffraction data are available at the Integrated Resource for Reproducibility in Macromolecular Crystallography (https://proteindiffraction.org) under the following DOIs: 10.18430/m37mhf, 10.18430/m37mhg, 10.18430/m37mhh, 10.18430/m37mhi, 10.18430/m37mhj, and 10.18430/m37mhk.

## 6. Glossary

*F*_o_ − *F*_o_ = isomorphous difference electron-density map. COVID-19 = coronavirus disease 2019 ▸. SARS-CoV-2 = severe acute respiratory syndrome coronavirus 2. M^pro^ = SARS-CoV-2 coronavirus main protease.

## Supplementary Material

PDB reference: SARS-CoV-2 M^pro^ multiconformer model, 7mhf

PDB reference: 7mhg

PDB reference: 7mhh

PDB reference: 7mhi

PDB reference: 7mhj

PDB reference: 7mhk

PDB reference: ensemble model, 7mhl

PDB reference: 7mhm

PDB reference: 7mhn

PDB reference: 7mho

PDB reference: 7mhp

PDB reference: 7mhq

Supporting information file. DOI: 10.1107/S2052252522007497/be5291sup1.pdf

URL: https://search.datacite.org/works/10.18430/m37mhf

URL: https://search.datacite.org/works/10.18430/m37mhg

URL: https://search.datacite.org/works/10.18430/m37mhh

URL: https://search.datacite.org/works/10.18430/m37mhi

URL: https://search.datacite.org/works/10.18430/m37mhj

URL: https://search.datacite.org/works/10.18430/m37mhk

## Figures and Tables

**Table 1:** Crystallographic statistics for multitem­per­ature data sets and multiconformer models Overall statistics given first (statistics for highest-resolution bin in parentheses). RH = relative humidity. RMSD = r.m.s. deviation from ideal values. For *Phenix* ensemble model refinement statistics, see Table 2 ▸.

Structure	100 K	240 K	277 K	298 K	298 K, 99.5% RH	310 K
PDB entry	7mhf	7mhg	7mhh	7mhi	7mhj	7mhk
Resolution (Å)	48.07–1.55	55.62–1.53	48.96–2.19	56.29–1.88	56.30–2.00	43.97–1.96
Completeness (%)	99.7 (96.0)	100 (99.4)	99.9 (98.7)	100 (100)	99.0 (97.4)	99.9 (100)
Multiplicity	3.4 (3.4)	6.6 (6.2)	6.9 (6.9)	6.8 (6.9)	6.8 (6.7)	6.6 (6.7)
*I*/σ(*I*)	3.3 (1.0)	7.9 (0.9)	4.5 (1.1)	5.0 (0.4)	6.3 (0.6)	5.4 (0.3)
*R*_merge_(*I*)	0.158 (0.507)	0.180 (1.463)	0.292 (1.805)	0.182 (2.353)	0.178 (1.708)	0.195 (1.805)
*R*_meas_(*I*)	0.188 (0.604)	0.196 (1.600)	0.316 (1.954)	0.197 (2.548)	0.193 (1.854)	0.213 (1.957)
*R*_p.i.m._(*I*)	0.100 (0.325)	0.076 (0.639)	0.119 (0.742)	0.076 (0.967)	0.074 (0.711)	0.084 (1.046)
CC_1/2_	0.977 (0.695)	0.995 (0.356)	0.985 (0.799)	0.990 (0.285)	0.989 (0.376)	0.990 (0.352)
Wilson *B* factor	16.164	16.370	31.769	29.670	34.350	33.810
Total observations	127548	263470	97820	152368	125878	128140
Unique observations	37901	39975	14120	22459	18588	19444
Space group	*C*121	*C*121	*C*121	*C*121	*C*121	*C*121
Unit-cell dimensions (Å, °)	113.71, 53.32, 44.57, 90, 102.96, 90	114.19, 53.49, 45.00, 90, 103.04, 90	115.02, 54.36, 44.97, 90, 101.50, 90	114.74, 54.57, 45.11, 90, 101.65, 90	114.88, 54.74, 45.24, 90, 101.42, 90	114.3, 54.29, 44.97, 90, 102.12, 90
Solvent content (%)	35.88	36.40	38.95	39.53	39.89	38.36
*R*_work_	0.1821	0.1692	0.1991 ▸	0.1906 ▸	0.1947 ▸	0.1979 ▸
*R*_free_	0.2242	0.2050 ▸	0.2525	0.2276	0.2397	0.2473
RMSD bonds (Å)	0.010	0.014	0.002	0.007	0.002	0.012
RMSD angles (°)	0.962	1.234	0.464	0.649	0.502	0.840
Ramachandran outliers (%)	0.00	0.33	0.33	0.33	0.33	0.66
Ramachandran favored (%)	97.70	98.36	96.38	96.71	96.38	97.04
Clashscore	2.80	1.61	1.68	1.68	0.84	1.68
*MolProbity* score	1.13	0.91	1.16	1.13	1.00	1.09

**Table 2:** Refinement statistics for *Phenix* ensemble models p_TLS_ and w_X-ray_ are input parameters to *Phenix* ensemble refinement; the other input parameter (τ_x_) was automatically determined (see *Methods* section).

Structure	100 K	240 K	277 K	298 K	298 K, 99.5% RH	310 K
PDB entry	7mhl	7mhm	7mhn	7mho	7mhp	7mhq
Resolution (Å)	1.55	1.53	2.19	1.88	2.00	1.96
p_TLS_	0.8	0.8	0.8	0.8	0.8	0.8
w_X-ray_	10.0	10.0	10.0	10.0	10.0	10.0
No. models in ensemble	54	43	45	75	28	36
*R*_work_	0.1658	0.1575	0.1531	0.1499	0.1594	0.1705
*R*_free_	0.2272	0.1967 ▸	0.2153	0.2083 ▸	0.2213	0.2348
