# Supplementary material for: The tem­per­ature-dependent conformational ensemble of SARS-CoV-2 main protease (Mpro)
Source: IUCrJ. 2022 Aug 17;9(Pt 5):682–94. doi: 10.1107/S2052252522007497 (PMC9438506; doi:10.1107/S2052252522007497)
Supplement: Supplementary file 1 [file m-09-00682-sup1.pdf]

IUCrJ

Volume 9 (2022)

Supporting information for article:

The temperature-dependent conformational ensemble of SARS-CoV-2 main protease (M^{pro})

Ali Ebrahim, Blake T. Riley, Desigan Kumaran, Babak Andi, Martin R. Fuchs, Sean McSweeney and Daniel A. Keedy

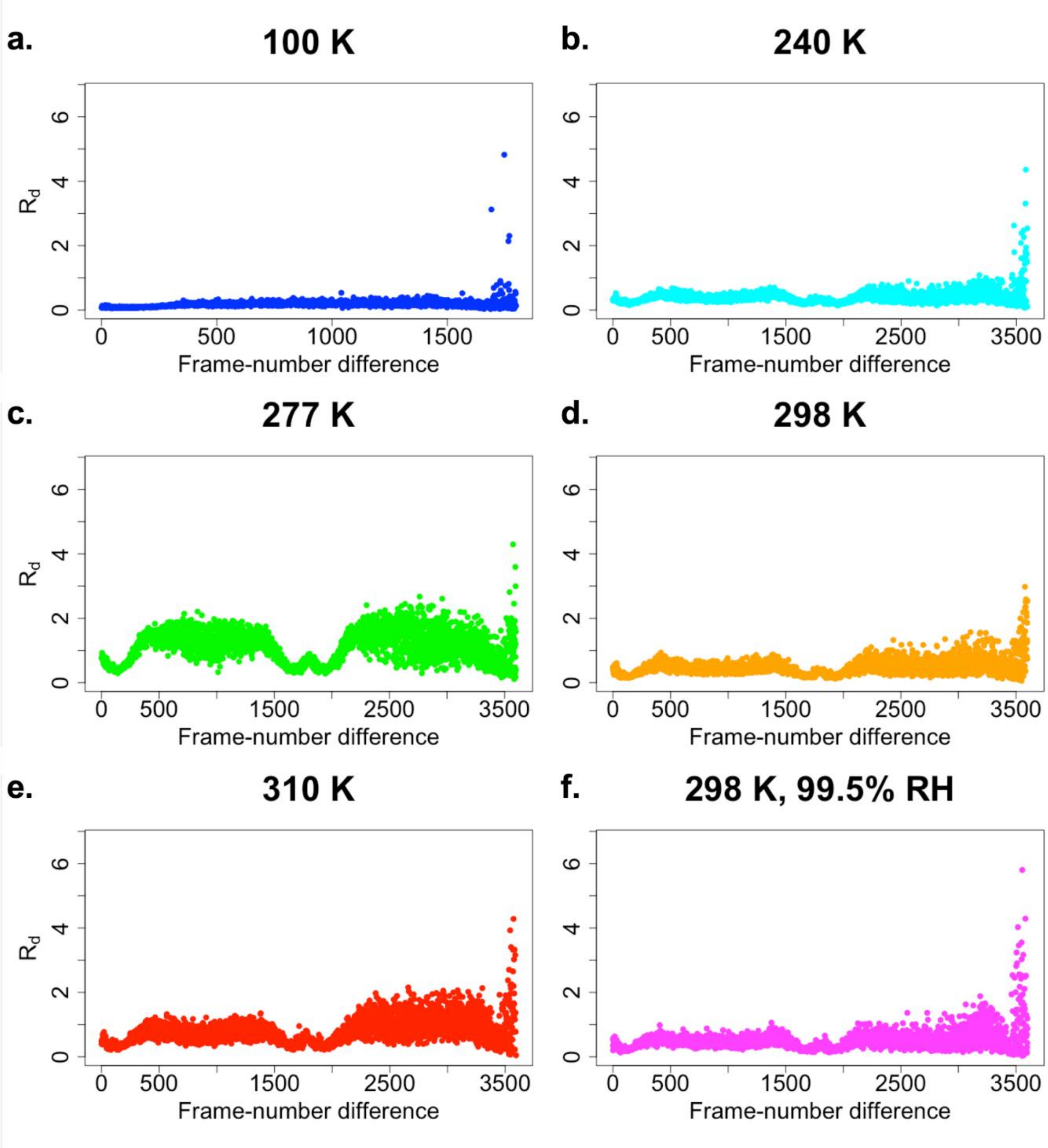

Fig. S1: Global radiation damage does not noticeably accrue during the course of each single-crystal data collection, as indicated by R_d as a function of frame-number difference. In addition, symmetric shapes of the plots derive from rotation of the thin, plate-like crystals during data collection, which unavoidably leads to different crystal volumes being irradiated for different frames; see **Fig. S11** for consideration of the effects of this diffraction anisotropy.

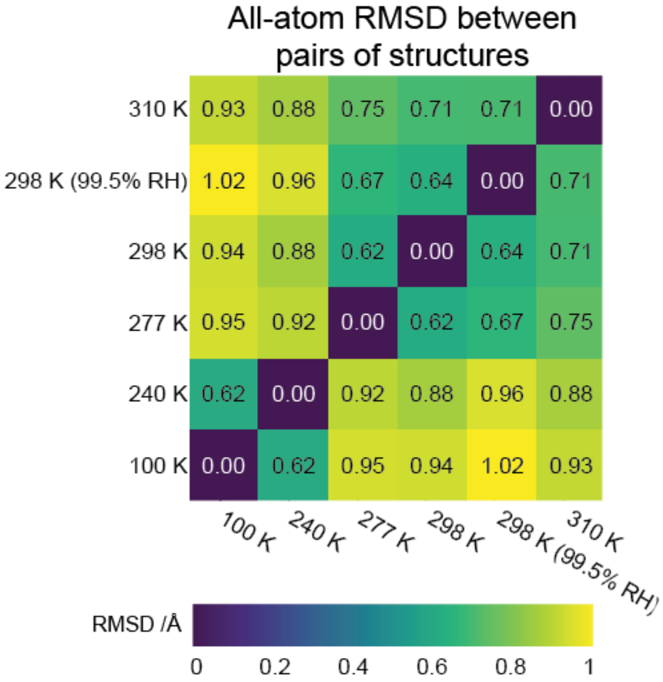

Fig. S2: Heatmap of pairwise all-atom RMSD between final refined structures, revealing temperature-dependent clustering (top-right vs. bottom-left).

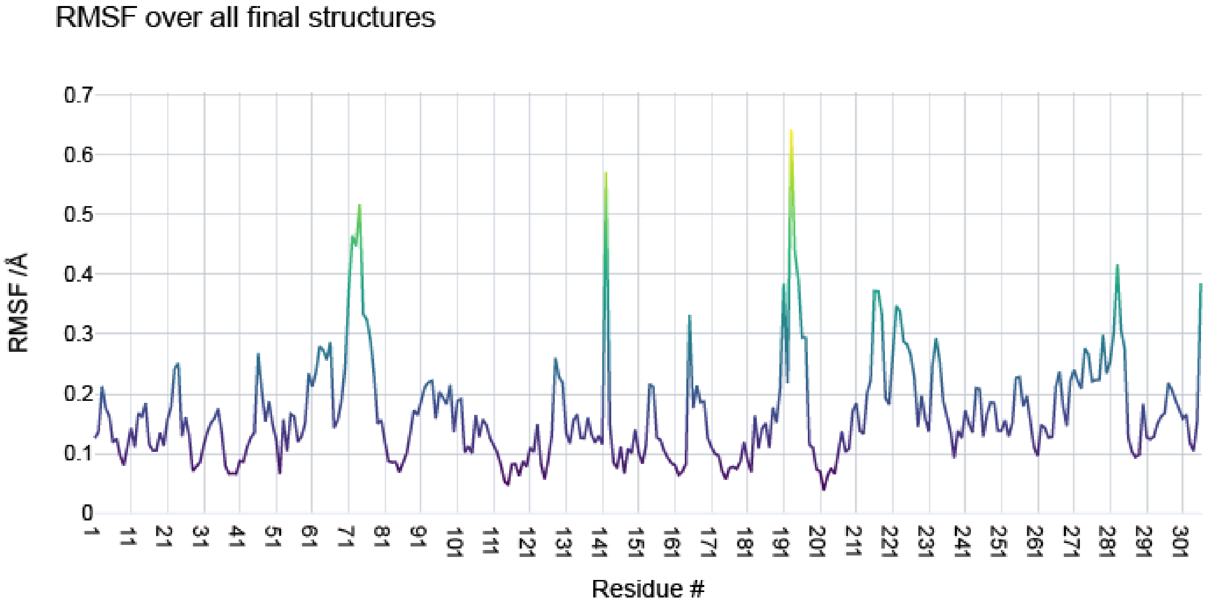

Fig. S3: Root-mean-square fluctuations (RMSF) of Cα atom positions over final refined structures. See also **Fig. 1c**.

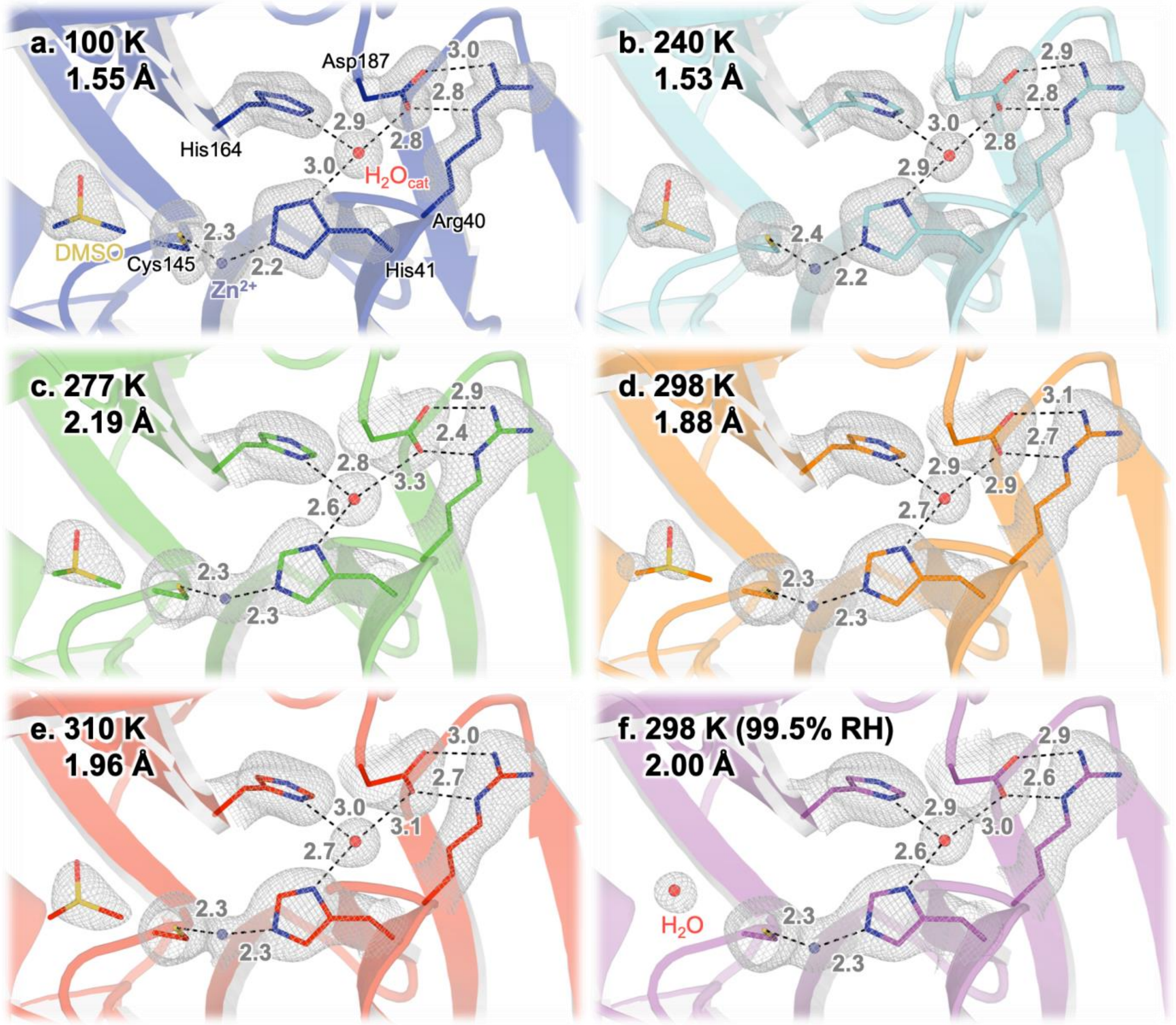

Fig. S4: The unliganded M^{pro} active site as a function of temperature and humidity.

$2F_o$-F_c electron density (1.0 σ, gray mesh) and interatomic distances (gray, in Å) shows that the active-site structure remains similar across datasets, including the catalytic dyad of His41 and Cys145 and the presumed catalytic water (H_2O_{cat}). One minor exception is an unanticipated low-occupancy Zn^{2+} ion which tends to shift upward in this view as temperature increases, adjusting its interactions with His41 and Cys145 (see also **Fig. S8**). An ordered DMSO molecule from the crystallization solution is visible at the left of each panel, except for 298 K at high humidity (99.5% RH) in which case a water is present at the same site instead. See also **Fig. S5**.

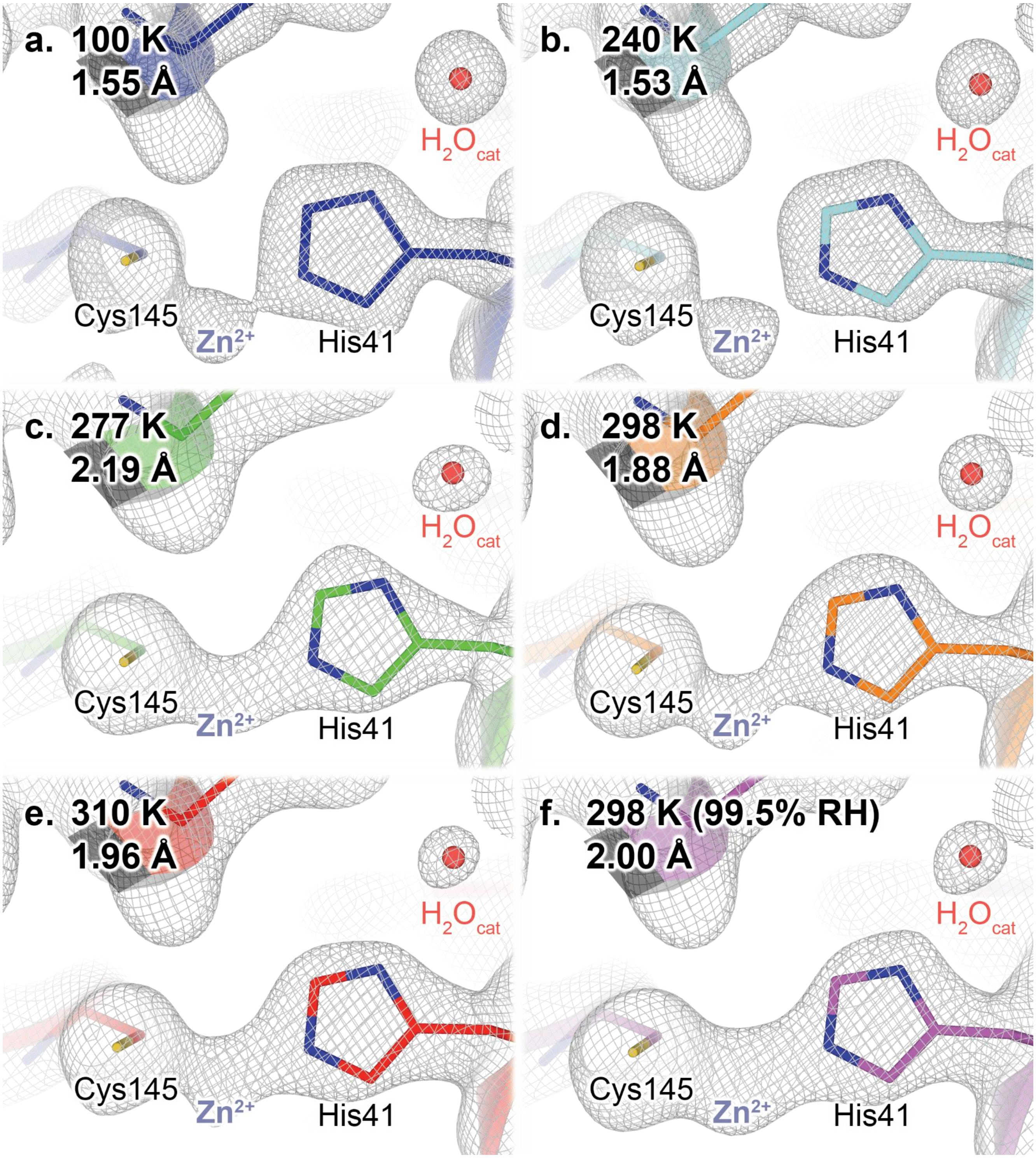

Fig. S5: Composite omit maps centered on the active site, contoured at 1 σ, for the structures reported in this work. A region of density is observed between His41 and Cys145.

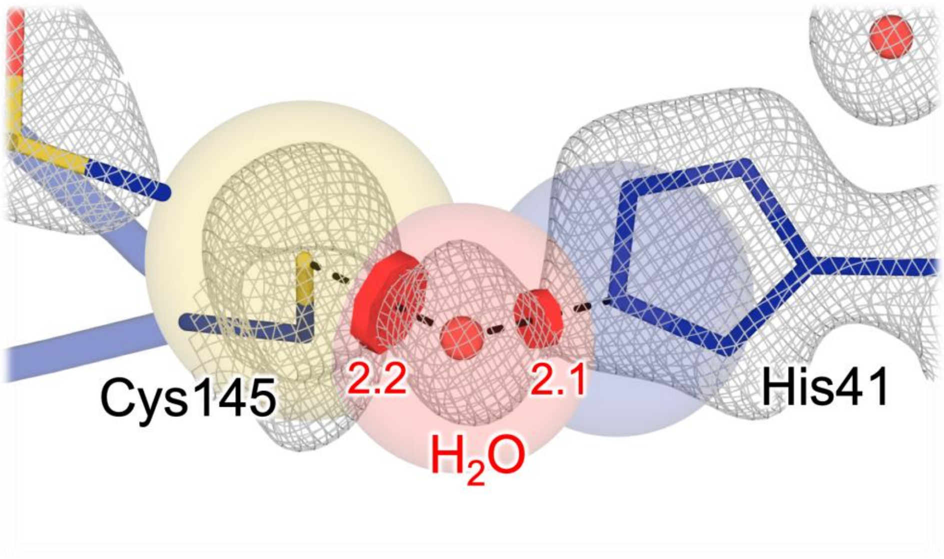

Fig. 6: Steric clashes if the peak amidst the catalytic dyad is modeled as H_2O instead of Zn^{2+}. Steric clashes are shown as red disks, with van der Waals overlaps shown as transparent spheres. Interatomic distances in Å. Steric clashes were visualized using the PyMOL show_bumps function.

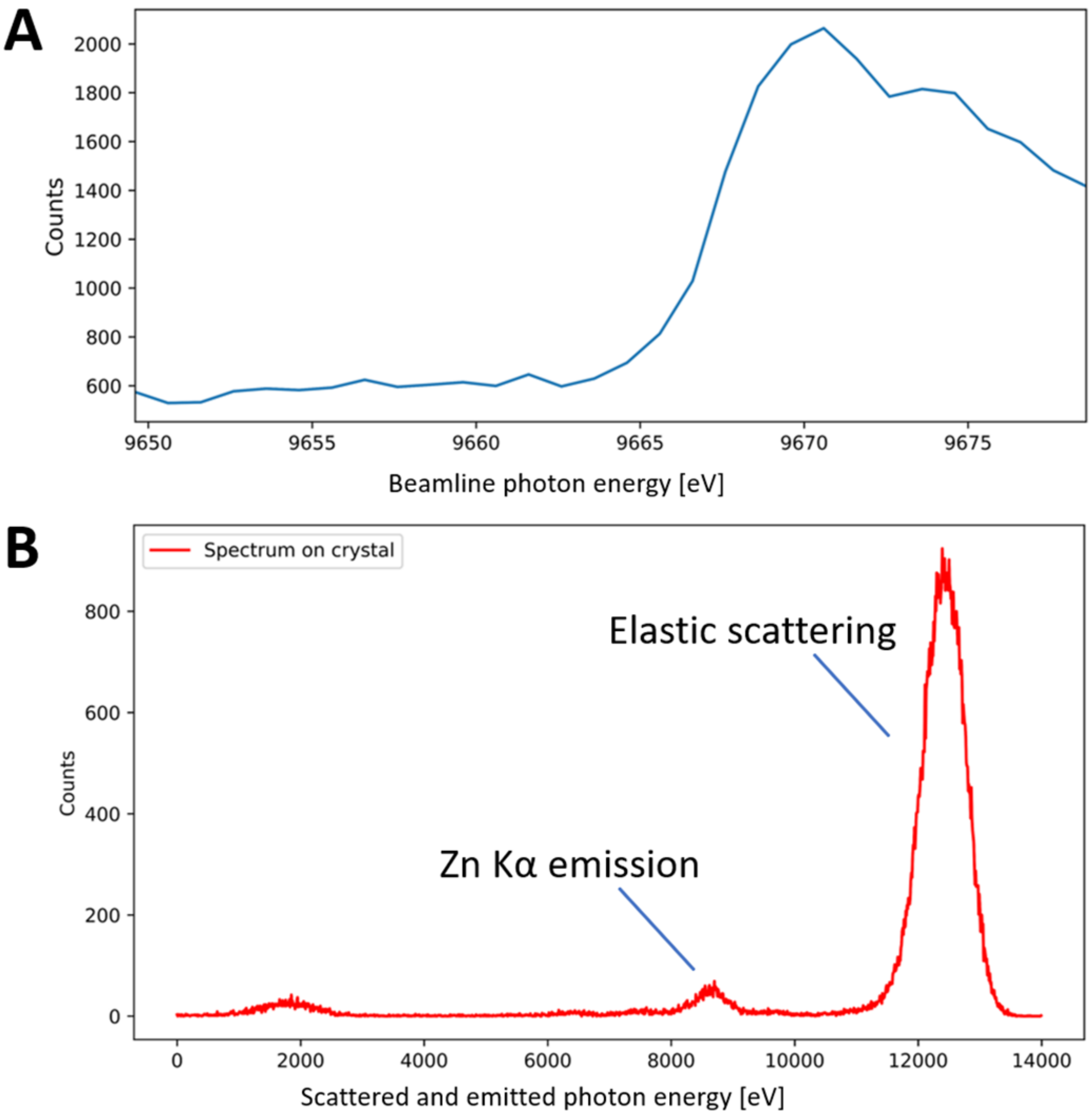

Fig. S7: a. X-ray fluorescence energy scan of an M^{pro} crystal at the Zn absorption edge. X-axis: incoming X-ray photon energy. Y-axis: emitted X-ray fluorescence photons at the Zn Ka emission line around 8639 eV. The increase of the intensity of the Zn specific emission when crossing the Zn absorption edge proves the presence of Zn in the crystal, either localized, or in the buffer and solvent channels. **b.** With an excitation photon energy of 12.66 keV, no other significant fluorescence emission besides the Zn line was observed, specifically not for other candidate metals such as Ni (expected Ka emission at 7.478 keV). The measured crystal was harvested from the same crystallization batch as the crystals used for multitemperature diffraction data collection.

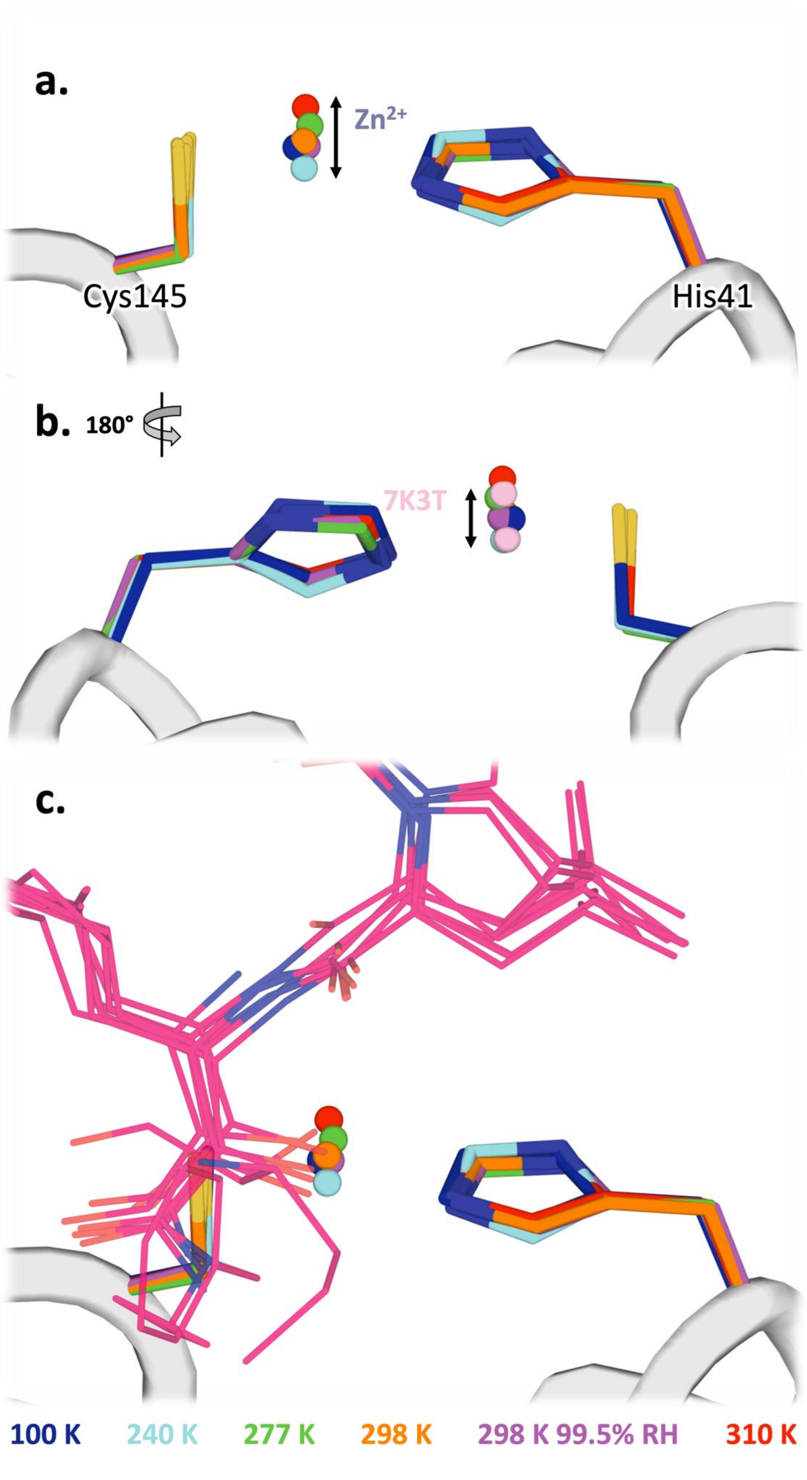

Fig. S8: A partial-occupancy zinc atom can access a swath of positions and is mimicked by ligands.

a. In our new structures, a partial-occupancy Zn^{2+} is ordered between Cys145 and His41 of the M^{pro} catalytic dyad. Its position varies as a function of temperature (blue to red) along a mostly linear swath. 298* K = 298 K at 99.5% relative humidity. The position of the Zn^{2+} is extremely similar at 298 K whether at ambient relative humidity or at 99.5% relative humidity.

b-c. The partial-occupancy Zn^{2+} swath in our structures is matched or mimicked by particular atoms in other previous structures of M^{pro}.

b. In 7K3T version 2.0, Zn^{2+} is modeled with two alternate conformations (pink), both of which are positioned along the swath we observe. The view is rotated 180° to better see the 7K3T Zn^{2+} atoms.

c. In several reported M^{pro} structures (6XFN, 6XBG, 6Y2G, 7NBR, 7K40, 7JYC) with covalent ligands (pink) linked to Cys145, a hydroxyl oxygen of the covalent adduct matches the position of the partial-occupancy Zn^{2+} swath in our structures. One of these thiohemiketals is observed in a distinct *(R)* conformation (6XFN), which places the hydroxyl oxygen at a more extreme position corresponding to the Zn^{2+} in our 310 K structure. The remainder of the ligands extend upwards into the substrate-binding pocket and are not visible in this image.

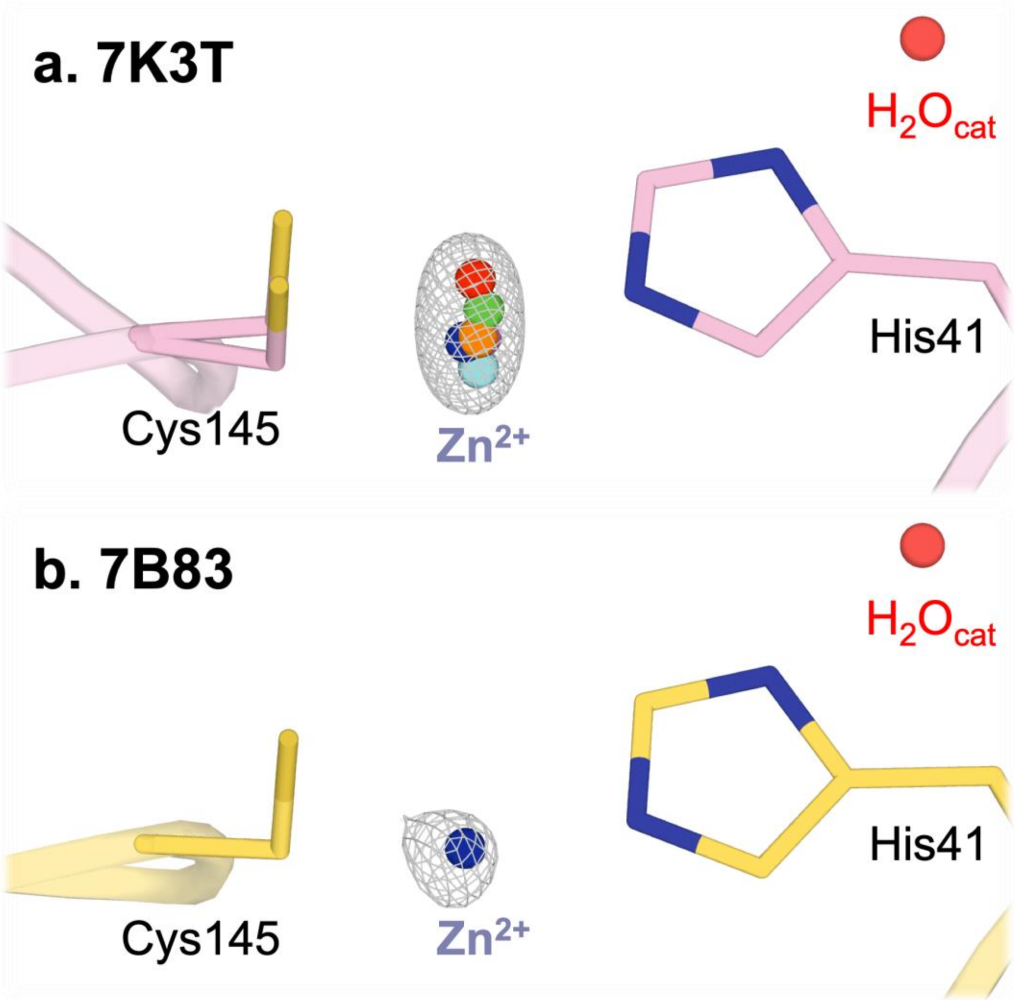

Fig. S9: Composite omit maps centered on the active site, contoured at 2.4 σ, for previously reported structures of SARS-CoV-2 M^{pro} with Zn^{2+} modeled in the active site. Here, that Zn^{2+} was removed and an omit map was calculated. In each case, a region of density is observed between His41 and Cys145 that is similar in position (although not necessarily strength) as in **Fig. S5**.

a. The swath of Zn^{2+} active site positions in our multitemperature series (coloring as in **Fig. S8**) can be superimposed directly into the composite omit active site density in the endogenous zinc bound structure 7K3T version 2.0.

b. The catalytic dyad of model 7B83 also binds zinc, though in this instance as a constituent of a zinc ionophore ligand. Zn^{2+} from our new 100 K model can be superimposed directly into the zinc composite omit density from 7B83.

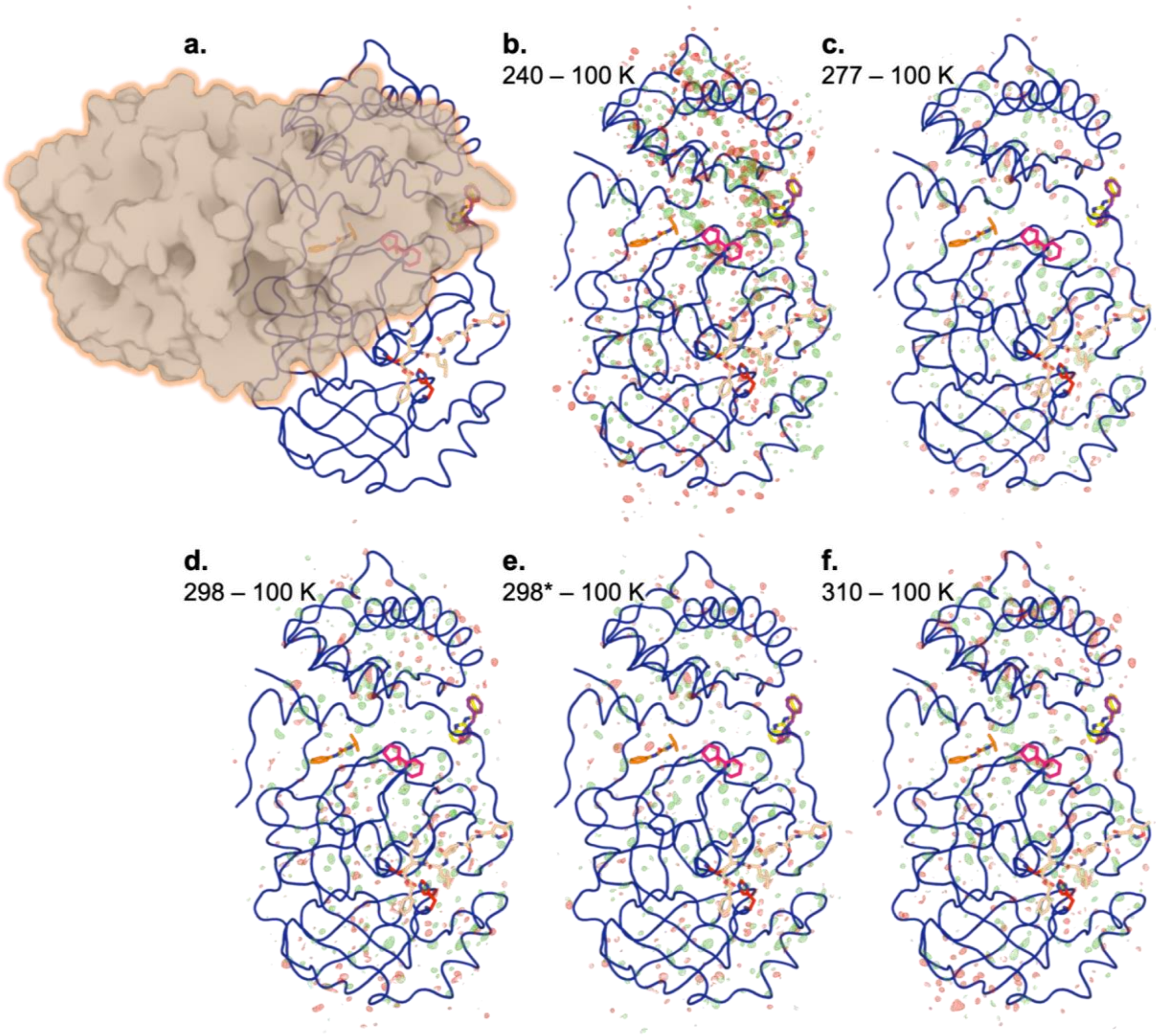

Fig. S10: Isomorphous F_o-F_o difference electron density maps for all elevated temperatures.

Ligands from cocrystal structures are shown as sticks at the active site (pale orange, 6LU7), inter-domain interface (purple, 5REE; yellow, 5REC), and dimer interface (orange, 7LFP; pink, 5FR0).

a. Overview of 100 K structure with superimposed ligands, and highlighted dimer interface (orange) (see **Fig. 5**).

b-f. F_o-F_o difference maps (+/-3 σ, green/red) are shown for each temperature minus 100 K.

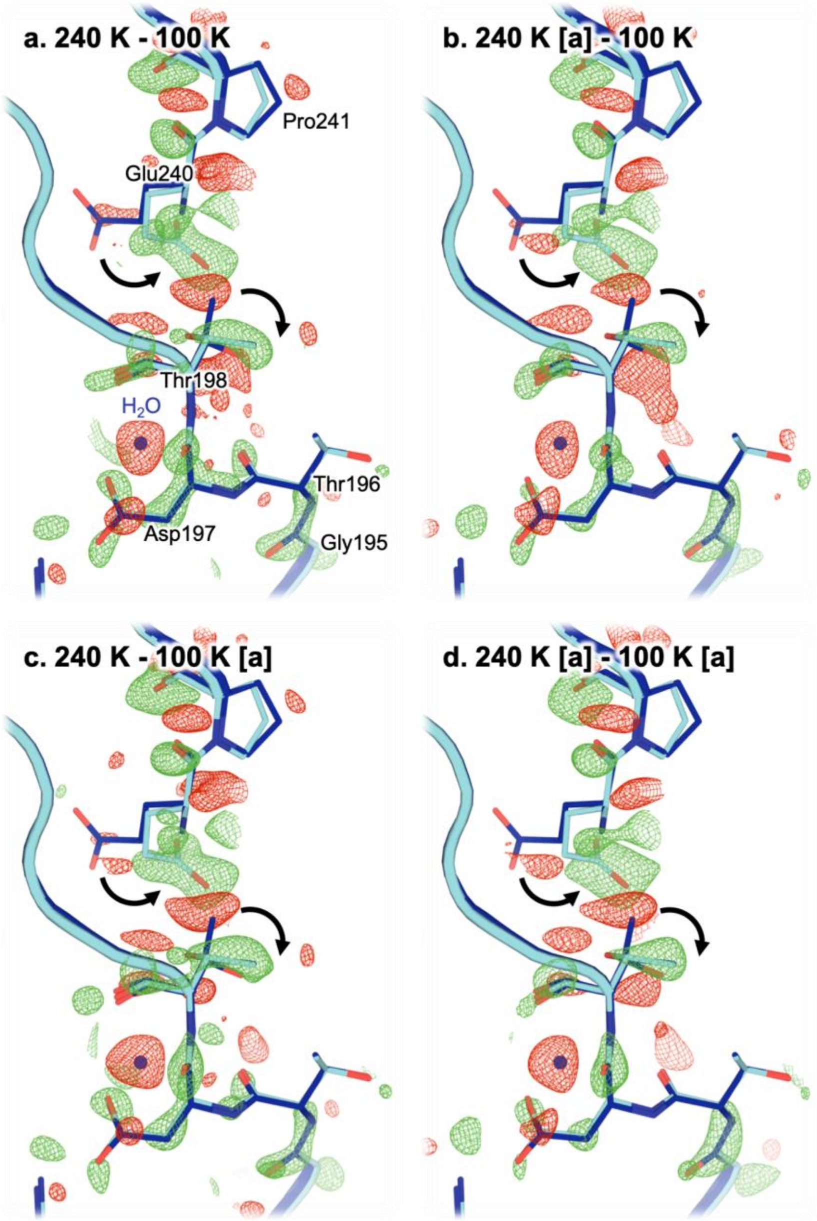

Fig. S11: Isomorphous F_o-F_o difference electron density maps involving anisotropically cut datasets at the P5 pocket region.

a. The isomorphous F_o-F_o difference density for the P5 binding pocket shown in **Fig. 3**. Changes to side-chain rotamers (curved arrows) are apparent as identified by areas of positive / negative difference density, contoured at +/- 3 σ (see **Fig. 5** for more detailed information).

b-d. Isomorphous F_o-F_o difference density when subtracting datasets that have been cut using anisotropic diffraction limits (datasets cut using the STARANISO server, denoted as “[a]") depict minimal change to the shape, position, and intensity of difference density.

d. When comparing datasets that have both been subjected to anisotropic diffraction limits, the effect of the dual to single conformation switch at Thr198 between 100 K and 240 K datasets, as well as the coinciding change in side-chain rotamer at Glu240, are still apparent.

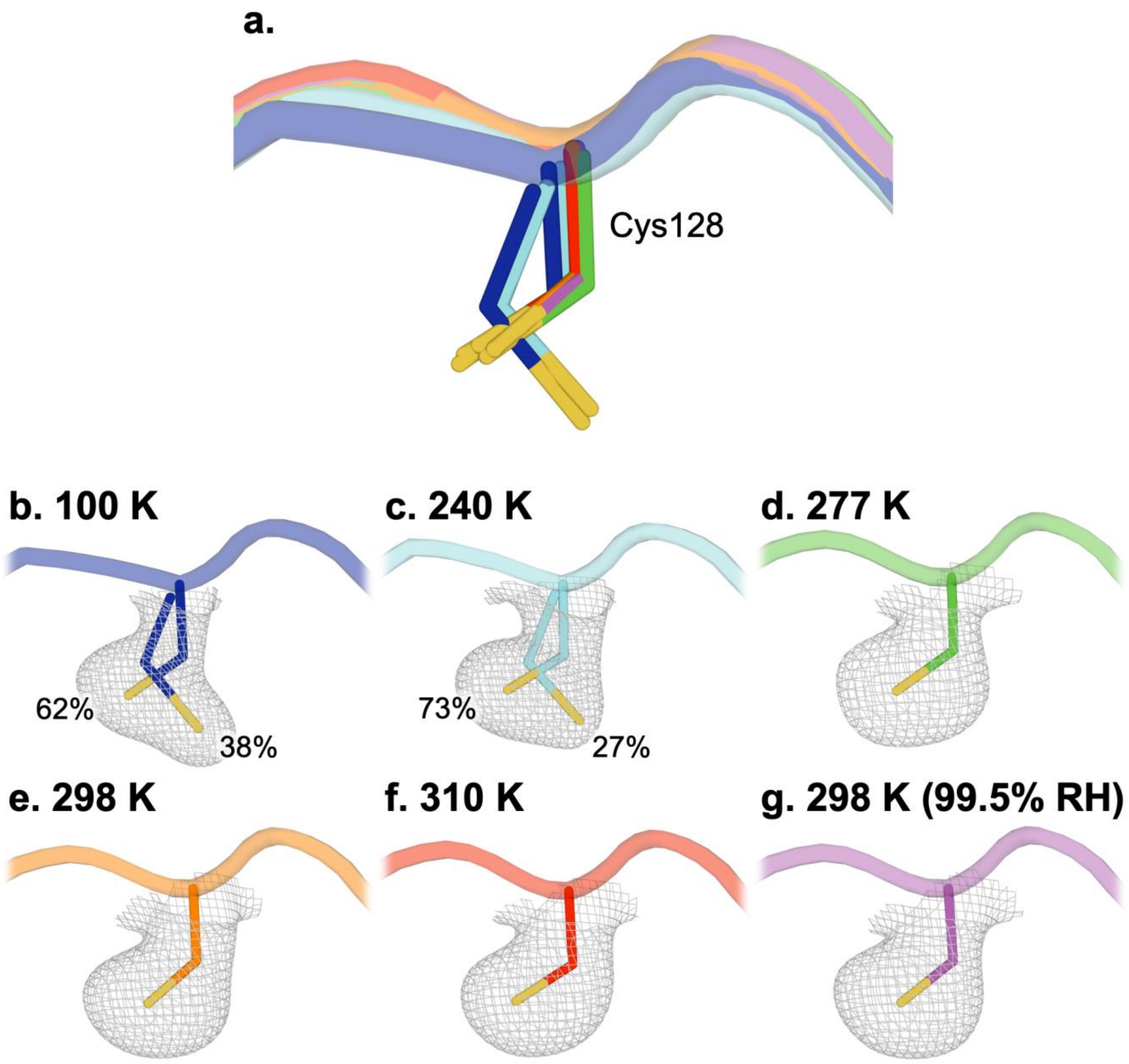

Fig. S12: Alternate conformations of dimer interface residue Cys128.

Similar viewing angle as in **Fig. 5a**.

a. Dual and single conformations of Cys128 shown as a superposition of all temperature series models. 100 K and 240 K models exist in dual conformation, while datasets collected at temperatures greater than this exist only in single conformation.

b-g. $2F_o$-F_c electron density maps highlighting dual and single conformations throughout the temperature series, contoured at 1 σ.

b. The 100 K dataset exhibits strong $2F_o$-F_c electron density for the Cys128 alternate rotamer, exhibiting a dual occupancy of 62% vs 38%, respectively.

c. As temperature is elevated, the occupancy of the alternate rotamer decreases to 27%, reducing the prominence of $2F_o$-F_c electron density in this region.

d-g. At temperatures of 277 K and above, Cys128 exists only as a single conformer residue.

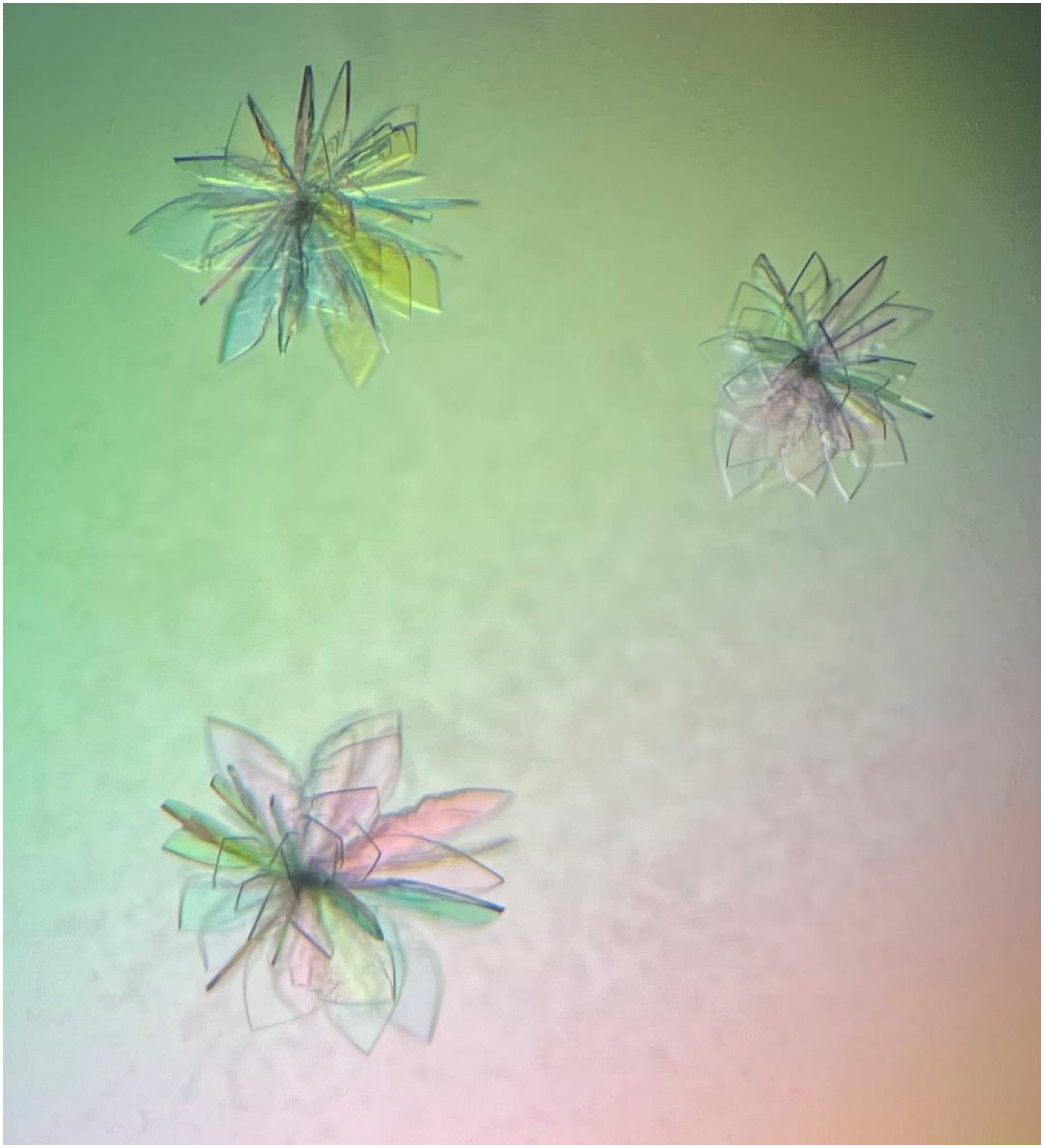

Fig. S13: Flower-like clusters of M^{pro} crystals. For each dataset, one “flower petal” (crystal) was manually harvested and used for X-ray diffraction. Each petal is approximately ~100–400 µm along the longest axis and ~5–10 µm along the shortest axis.
